# STARD13-correlated ceRNA network-directed inhibition on YAP/TAZ activity suppresses stemness of breast cancer via co-regulating Hippo and Rho-GTPase/F-actin signaling

**DOI:** 10.1186/s13045-018-0613-5

**Published:** 2018-05-30

**Authors:** Lufeng Zheng, Chenxi Xiang, Xiaoman Li, Qianqian Guo, Lanlan Gao, Haiwei Ni, Yufeng Xia, Tao Xi

**Affiliations:** 1 School of Life Science and Technology, Jiangsu Key Laboratory of Carcinogenesis and Intervention, China Pharmaceutical University, Nanjing, 210009 China; 2 Jiangsu Key Laboratory of Drug Discovery for Metabolic Diseases, Department of Pharmacology of Chinese Materia Medica, China Pharmaceutical University, 24 Tong Jia Xiang, Nanjing, 210009 China; 3 Jiangsu Key Laboratory for Pharmacology and Safety Evaluation of Chinese Materia Medica, School of Pharmacy, Nanjing University of Chinese Medicine, Nanjing, 210023 China

**Keywords:** STARD13 ceRNA YAP/TAZ Hippo F-actin CSC breast cancer

## Abstract

**Background:**

Targeting cancer stem cells is critical for suppressing cancer progression and recurrence. Finding novel markers or related pathways could help eradicate or diagnose cancer in clinic.

**Methods:**

By constructing STARD13-correlated ceRNA 3′UTR stable overexpression or knockdown breast cancer cells, we aimed to explore the effects of STARD13-correlated ceRNA network on breast cancer stemness in vitro and in vivo. Further RNA-sequencing was used to analyze transcriptome change in combination with functional studies on candidate signaling. Clinical samples obtained from The Cancer Genome Atlas data were used to validate the correlation between STARD13 and related pathways. Finally, in vitro and in vivo experiments were used to examine the effects of STARD13-correlated ceRNA network on chemotherapy sensitivity/resistance.

**Results:**

Here, we revealed that this ceRNA network inhibited stemness of breast cancer. Mechanistically, we found that activation of STARD13-correlated ceRNA network was negatively correlated with YAP/TAZ activity in breast cancer. Specifically, this ceRNA network attenuated YAP/TAZ nuclear accumulation and transcriptional activity via collectively modulating Hippo and Rho-GTPase/F-actin signaling. Finally, we demonstrated that YAP/TAZ transcriptional activity regulated by this ceRNA network was involved in chemoresistance.

**Conclusions:**

Our results uncover a novel mechanism of YAP/TAZ activation in breast cancer and propose the possibility to drive STARD13-correlated ceRNA network to inhibit breast cancer stem cell traits.

**Electronic supplementary material:**

The online version of this article (10.1186/s13045-018-0613-5) contains supplementary material, which is available to authorized users.

## Background

Breast cancer is the most common cancer in women worldwide, and its incidence is increasing yearly [1]. At present, chemotherapy and surgery are the main methods for breast cancer treatment; especially, chemotherapy is the only option for triple negative breast cancer [2]. However, tumor relapse and chemoresistance constitute a major detriment to patients’ treatment and survival, and the mechanisms underpinning these phenomena remain elusive, which is an urgent need to solve. Since tumor relapse and chemoresistance are a complex network that integrates multiple growth control signals through an expanding set of core elements, a single factor could not make a good assessment of chemotherapy or tumor recurrence. In fact, we have only a scattered understanding of the molecular mechanisms that are responsible for tumor relapse and chemoresistance.

Cancer stem cells (CSCs) are proposed to drive tumor relapse and chemoresistance [3]. CSCs are a small group of cells within the tumor bulk, which remain dominant under the rapid growth of tumor cells, thus circumventing chemical agents, and then spawn non-CSC progeny, leading to tumor recurrence or chemoresistance [4]. It was proposed that in the course of malignant progression, the increased proportion of the CSCs within the tumor is inextricably twined with activation of the epithelial-to-mesenchymal transition (EMT) [5]. EMT is a complex transdifferentiation program that is instrumental for the acquisition of stemness by non-transformed tumor cells [6, 7]. Several genes involved in maintaining self-renewal capacity were also demonstrated to enhance EMT features [8]. Over the past several years, competing endogenous RNAs (ceRNAs) have emerged as an important class of post-transcriptional regulator that alters gene expression through an miRNA-mediated mechanism [9, 10]. We and others have revealed that ceRNAs have significant roles in cancer pathogenesis by altering the expression of key tumorigenic or tumor-suppressive genes in both solid tumors and hematopoietic malignancies [11–14]. Our recent work has confirmed that STARD13, CDH5, HOXD1, and HOXD10 could co-regulate each other through competing for several shared miRNA binding sites, and thus formed a ceRNA network to coordinately inhibit breast cancer EMT and metastasis, which is denoted as STARD13-correlated ceRNA network [15]. Here, we further seek to explore the mechanisms by which STARD13-correlated ceRNA network regulates EMT and its roles in breast CSC formation.

The mammalian Hippo pathway is a kinase cascade involving mammalian STE20-like protein kinase 1 (MST1) and the large tumor suppressor 1 (LATS1) and LATS2 [16]. LATS1/2, as the core elements of Hippo pathway, could phosphorylate and inactivate the downstream effectors YAP/TAZ that mediate transcriptional output of the Hippo pathway. A previous study has indicated that TAZ could confer CSC-related traits on breast cancer cells [17]. YAP/TAZ are considered as “stemness factors” in several types of stem cells [18]. Inhibition of YAP/TAZ activity could attenuate breast cancer EMT and invasion [19]. Notably, LATS1/2 could suppress breast cancer EMT and metastasis via inactivating YAP/TAZ activity [20]. In line with this, aberrant YAP/TAZ transcriptional activity is frequent in numerous tumors, making YAP/TAZ as a potential target for cancer treatment. However, efforts in this direction are frustrated by the fact that the Hippo cascade is largely undruggable [21]. While the core components of the Hippo signaling are well established, numerous additional upstream regulators of YAP/TAZ activity are emerging, such as the transcriptional activity of YAP/TAZ is influenced by cell mechanics, and this process is driven through Rho-GTPase/F-actin signaling, a manner largely independent on LATS1/2 [22]. In addition, actin remodeling factors control ciliogenesis by regulating YAP/TAZ activity [23]. Rho-GTPase/F-actin signaling could promote the long-term survival and expansion of human embryonic stem cells [24]. The fact that LATS1/2 and F-actin organization act independently to regulate YAP/TAZ is also supported by genetic evidence in *Drosophila* [25]. Despite the clear association of Rho-GTPase/F-actin and Hippo-YAP signaling in various cancers, targeted therapies aiming at these two pathways remain limited. Collectively, these findings speak to that coordinately activating Hippo signaling and inactivating Rho-GTPase/F-actin pathway might be an ideal way to suppress YAP/TAZ activity, and thus CSC formation.

Here, we found that STARD13-correlated ceRNA network suppressed breast CSC formation in vitro and in vivo. To characterize the mechanisms and roles of STARD13-correlated ceRNA network, we performed a candidate functional screen and identified LATS1/2 and RhoA/F-actin signaling as essential for STARD13-correlated ceRNA network-mediated inhibition on breast CSC formation. We further found that YAP/TAZ were the major downstream factors in this process. Finally, we indicated that STARD13-correlated ceRNA network enhanced doxorubicin sensitivity in breast cancer cells.

## Methods

### Cell culture

HEK293T cells and human breast cancer cells MCF-7 and MDA-MDB-231 were stored in our laboratory. Cell line authentication was assessed using short tandem repeat (STR) DNA profiling method every year. HEK293T and MCF-7 cells were cultured in DMEM medium (Gibco, Grand Island, NY, USA), and MDA-MB-231 cells were cultured in L-15 medium (Gibco) at 37 °C under a humidified atmosphere with 5% CO_2_. Both of the media were supplemented with 10% FBS (Gibco), 80 U/ml penicillin, and 0.08 mg/ml streptomycin.

### Cell transfection

Transfection of plasmids was performed using Lipofectamine 2000 (Invitrogen, Carlsbad, CA) on MCF-7 cells, TransIT-BrCa Transfection Reagent (Mirus, USA) on MDA-MB-231 cells, and Lentifection (ABM, Vancouver, Canada) on HEK293T cells. A final concentration of siRNA (GenePharma, China) was 50 nM. Sequences of siRNA against a specific target in this study were listed in Additional file 1: Table S1.

### RNA isolation and quantitative real-time PCR analysis

Total RNA was extracted by TRIZOL reagent (Invitrogen, USA) according to the manufacturer’s instructions. qRT-PCR was performed on triplicate samples in a reaction mix of SYBR Green (Vazyme, China) with Roche Real-Time PCR system (Roche, USA). mRNA and miRNA levels were normalized to GAPDH or U6 sRNA, respectively. The relative expression levels of indicated genes were calculated using 2^-ΔΔCt^ method. Sequences of primers used for qRT-PCR in this study were listed in Additional file 2: Table S2.

### Immunohistochemistry and immunohistofluorescence assays

Paraffin-embedded sections were deparaffinized and rehydrated, followed by antigen retrieval. After primary and secondary antibody incubation, the slide was finally incubated with diaminobenzidine (DAB) (Dako, USA) for IHC analysis and observed with the confocal microscopy.

### Immunofluorescence and F-actin visualization

The detailed procedure was referred to our previous study [26].

### RhoA GTPase assay

The detailed procedure was referred to our previous study [26].

### Western blot analysis

Protein lysates were obtained from cells grown for 48 h at high density. The Western blot procedure was carried out as described in our previous work [26]. The information of primary antibodies were listed in Additional file 3: Table S3.

### RNA immunoprecipitation

RNA immunoprecipitation (RIP) assays were conducted using the Protein A/G Agarose Resin 4FF (YEASEN, Shanghai, China) following the manufacturer’s protocol. Briefly, cells were lysed by NP-40 lysis buffer (Beyotime, China). Then, 100 μl cell lysates were incubated with NP-40 buffer containing Protein A/G Agarose Resin 4F conjugated with human anti-Ago2 antibody (Cell Signaling Technology) at 4 °C overnight. After that, agarose beads were isolated by centrifugation and incubated with protease K to dissociate Ago2-RNA complex from the beads. The RNA fraction precipitated by RIP was analyzed by qRT-PCR.

### In vivo tumor initiation and doxorubicin sensitivity assays

Four- to six-week male athymic BALB/c nude mice were purchased from Model Animal Research Center of Nanjing University and were housed and fed in standard pathogen-free conditions. For tumor-limiting dilution assays, tumor cells were mixed 1:1 with Matrigel matrix (BD Biosciences) and orthotopically implanted in the inguinal mammary gland of mice. On day 8, all mice were killed, and tumor tissues were collected, weighed, and fixed in 10% formalin at room temperature and embedded in paraffin for immunohistochemistry or immunohistofluorescence assay. For doxorubicin sensitivity assay, MCF-7 cells with STARD13 or its ceRNA stable knockdown or not and MDA-MB-231 cells with STARD13 3′UTR overexpression or not were injected subcutaneously into each flank of 5-week-old BALB/c female mice. When the tumors reached the volume of ~ 100 mm^3^, we randomly allocated the mice to groups in which they received doxorubicin (0.5 mg/kg, Shenzhen Main Luck Pharmaceuticals Inc., China) or saline. Tumor growth was monitored by caliper measurements. Tumor volume was calculated by the following formula: Volume (cubic millimeters) = L (length) × W (width)^2^ × 1/2. Mice were euthanized 18 days after the inoculation. The weight of each tumor was measured.

### Plasmid and stable expression cell line constructions

For stable expression of STARD13-3′UTR, CDH5-3′UTR, HOXD1-3′UTR, and HOXD10-3′UTR, sequences of 3′UTRs were subcloned into pLVX-ZsGreen and referred as pLVX-ceRNAs-3′UTR. shRNA oligos were synthesized by Sangon Co., Ltd. After annealing, double-strand oligos were inserted to lentiviral pLKO.1-Puro vector (Addgene). To package lentivirus, HEK-293T cells were co-transfected with the lentiviral vector described above and packaging vectors psPAX2 and pMDG.G using Lentifectin (ABM, USA). Cells were infected with the virus in the presence of 2 μg/ml polybrene. The infected cells were selected with puromycin (Sigma, 2 μg/ml) for 2 weeks. After two rounds of infection, qRT-PCR and Western blot analyses were used for verification. Meanwhile, cells infected with pLVX-ceRNAs-3′UTR were selected by fluorescent cell sorting.

The 3′UTRs of LATS1 and LATS were cloned into the luciferase reporter vector (pMIR-Report, Ambion, Carlsbad, CA, USA), and the corresponding plasmids were denoted as pMIR-LATS1-3′UTR and pMIR-LATS2-3′UTR. Sequences of primers used for plasmid construction in this study were listed in Additional file 4: Table S4. All constructs were confirmed by DNA sequencing.

### Luciferase reporter assay

Cells were co-transfected with pMIR luciferase reporter or β-gal and siRNA using Lipofectamine 2000. Each group was run in triplicate in 96-well plates. The luciferase activity was detected by Luciferase Reporter Assay System (Promega, USA) after 48 h of transfection. Luciferase activity was normalized to β-gal activity.

### Flow cytometric assay

Cells were detached from plates with Accutase (Invitrogen), resuspended (1 × 10^6^ cells/ml), incubated in running buffer (PBS 1×, BSA 0.5%, and EDTA 5 mM) with anti-human CD44 (APC-conjugated, BD Biosciences) and anti-human CD24 (PE-conjugated, BD Biosciences), and finally analyzed on a C6 flow cytometer (BD Biosciences). Flow cytometry values have been normalized by subtracting the appropriate isotype control value.

### Mammosphere formation assay

Cells were grown in the MammoCult medium (Stem Cell Technologies, Vancouver, Canada) supplemented with MammoCult Proliferation Supplements (Stem Cell Technologies, Vancouver, Canada) and plated in 24-wells plate with ultra-low attachment at a density of 10,000 viable cells/ml and grown for 10 days. Mammospheres were counted and photographed.

### RNA sequencing and data analysis

RNA from MDA-MB-231 and STRARD13 3′UTR-overexpressed MDA-MB-231 cells (MDA-MB-231 (S-UTR)) was extracted using TRIzol. RNA-seq libraries were acquired using TruSeq PE Cluster Kit V4 and sequenced by HiSeq 2000 sequencer. We used Cuffdiff to estimate fragments per kilobase of transcript per million (FPKM) values for known transcripts and analyze differentially expressed transcripts. *p* < 0.05 was considered as significant. Heatmap of gene expression was generated based on log2 (FPKM) using HemI 1.0.3.7 (http://hemi.biocuckoo.org/down.php). GO analysis of gene expression changes was performed using GO-TermFinder. KEGG enrichment analysis was performed according to Rich factor, Qvalue, and numbers of enriched genes. The richer factor is bigger, and the degree of enrichment is bigger; the Qvalue is smaller, and the degree of enrichment is more significant.

### Patient samples

Paired mRNA profiling data were downloaded from The Cancer Genome Atlas (TCGA) data portal (http://cancergenome.nih.gov). The dataset from the Mixed Tumor Breast – Clynes – 121 – MAS5.0 – u133p2 public (http://hgserver1.amc.nl/cgi-bin/r2/main.cgi), which includes 121 breast cancer samples, was obtained as a validation set. The microarray dataset was deposited in the Gene Expression Omnibus (GEO) (accession number GSE42568) according to “minimum information about a microarray experiment” (MIAME) guidelines. The R2 platform was used to analyze the microarray (http://r2.amc.nl).

### Statistical analysis

All data were obtained from at least three independent experiments (*n* ≥ 3) and presented as the mean ± SD (standard deviation). Statistical analyses were performed using Student’s *t* test except for qRT-PCR. Data from the qRT-PCR test were analyzed using one-way analysis of variance (ANOVA). The differences between the groups were analyzed using ANOVA with the Tukey-Kramer post-test. *p < 0.05 was considered statistically significant. **p < 0.01, ***p < 0.001, and ns indicate no significant differences from control.

## Results

### STARD13-correlated ceRNA network inhibits the stemness-related traits in breast cancer cells

**Fig. 1:**
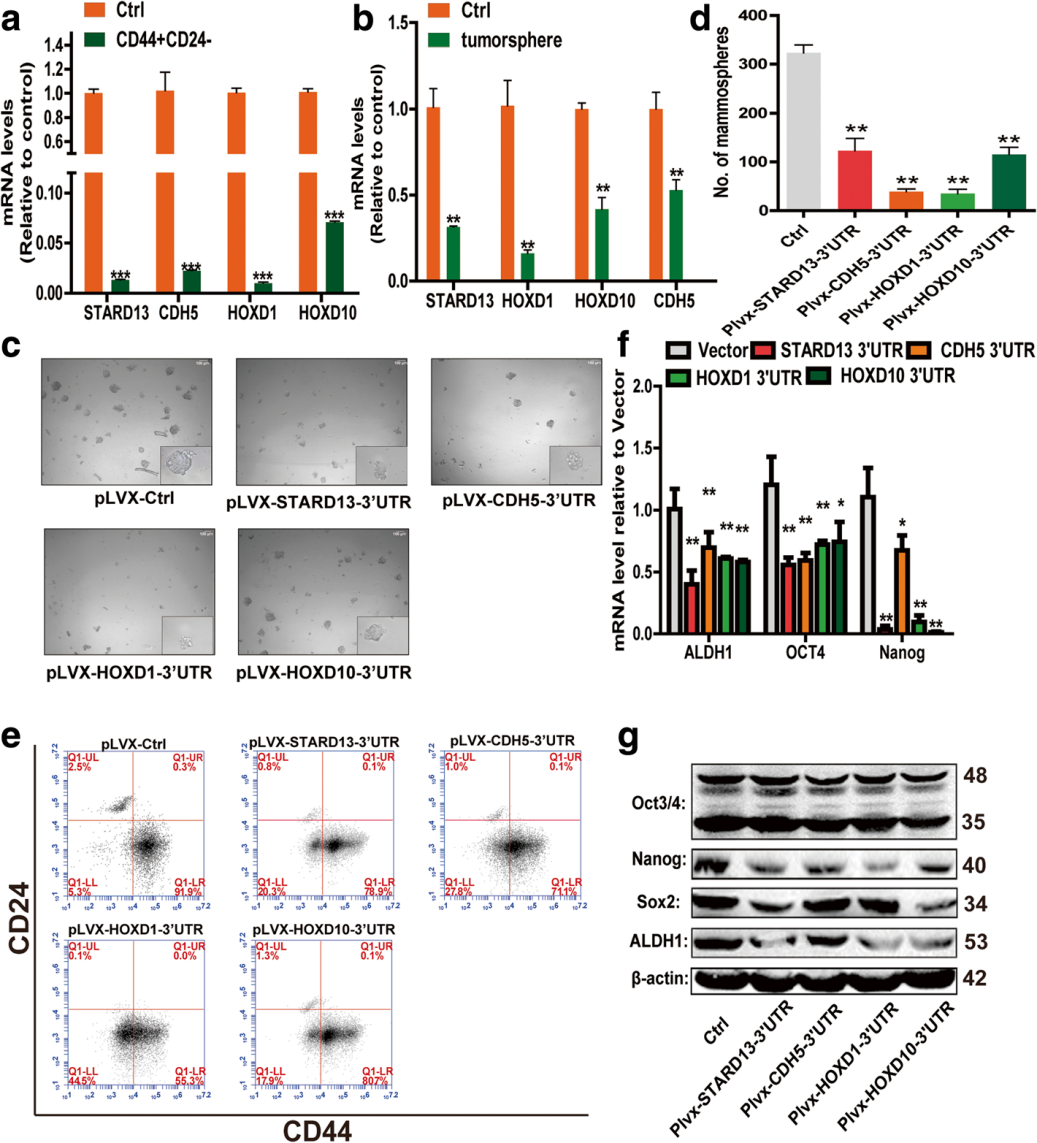
STARD13-correlated ceRNA network inhibits tumor sphere formation capacity of breast cancer in vitro. **a**, **b** Detection of STARD13, CDH5, HOXD1, and HOXD10 expressions in sorted CD44+/CD24− cell subpopulation (**a**) and mammospheres (**b**) of MCF-7 cells enriched in 3D semi-solid culture medium by qRT-PCR analysis. (**c**) Phase contract images of mammospheres formed by MDA-MB-231 derivatives (MDA-MB-231 stably overexpressing with STARD13 3′UTR, CDH5 3′UTR, HOXD1 3′UTR, and HOXD10 3′UTR, and their negative control, hereafter indicated as pLVX-STARD13-3′UTR, pLVX-CDH5-3′UTR, pLVX-HOXD1-3′UTR, pLVX-HOXD10-3′UTR, and pLVX-Ctrl, respectively). **d** Quantification of mammospheres in **c**. **e** Representative FACS profile of MDA-MB-231 derivatives described in **c** with CD24− and CD44+ markers. **f**, **g** Examination of stemness-related genes expression (ALDA1, OCT4, and Nanog) by qRT-PCR (**f**) and Western blot analysis (**g**) in cells described in **c**. Data were presented as the mean ± SD, *n* = 3, *p < 0.05, **p < 0.01, ***p < 0.001 vs. vector or control

To investigate whether STARD13-correlated ceRNA network is involved in breast CSC formation, we first detected the expression levels of STARD13, CDH5, HOXD1, and HOXD10 in a pool of CSCs naturally arising within breast cancer cells. Fluorescence-activated cell sorting (FACS) was used to sort MCF-7 cells based on the expressions of cell surface antigen markers CD44+/CD24−, which have been identified as the breast CSC markers [17]. As shown in Fig. 1a, the qRT-PCR analysis revealed that a CD44+/CD24− subpopulation displayed lower levels of STARD13-correlated ceRNAs. As previous studies have demonstrated that non-adherent spheres are highly enriched for CSCs [27, 28], the expression levels of STARD13-correlated ceRNAs were examined in non-adherent spheres and parental cells, and an identical result was acquired (Fig. 1b). We next tested whether STARD13-correlated ceRNAs-3′UTR overexpression could impair the capacity of tumorsphere formation. MDA-MB-231 cells stably transfected with STARD13-correlated ceRNAs-3′UTRs or an empty vector were subjected to non-adherent sphere formation assay. The infection efficiency of lentivirus was verified by qRT-PCR assay (Additional file 5: Figure S1A). Both the sphere size and number were significantly decreased after STARD13-correlated ceRNAs-3′UTR overexpression (Fig. 1c, d). Additionally, overexpression of STARD13-correlated ceRNAs-3′UTRs decreased the CD44+/CD24− population by flow cytometry analysis (Fig. 1e). Moreover, the expression of several pluripotent transcription factors, namely, Oct3/4, ALDH1, Nanog, and Sox2, was decreased in cells with STARD13- or its ceRNAs-3′UTR overexpression (Fig. 1f, g). We further tested whether the knockdown of STARD13-correlated ceRNAs could confer stemness properties in MCF-7 cells. Western blot assay confirmed the knockdown efficiency of shRNAs against STARD13-correlated ceRNAs (Additional file 5: Figure S1B). Functionally, the knockdown of STARD13-correlated ceRNAs formed more primary mammospheres than control cells (Additional file 6: Figure S2A and S2B) and induced an enlargement of the CD44+/CD24− population (Additional file 6: Figure S2C). As expected, the expression of stemness markers was upregulated in cells with STARD13-correlated ceRNA knockdown (Additional file 6: Figure S2D). Since we found that the CD44+/CD24− subpopulation is more than 90% in MDA-MB-231 cells, and less than 20% in MCF-7 cells, this phenomenon indicated that MDA-MB-231 and MCF-7 cells held high CSC-related traits and low CSC-related traits, respectively. Thus, MDA-MB-231 and MCF-7 cells were used for overexpression and knockdown experiments, respectively.

### STARD13-correlated ceRNA network inhibits CSC traits of breast cancer cells in vivo

**Fig. 2:**
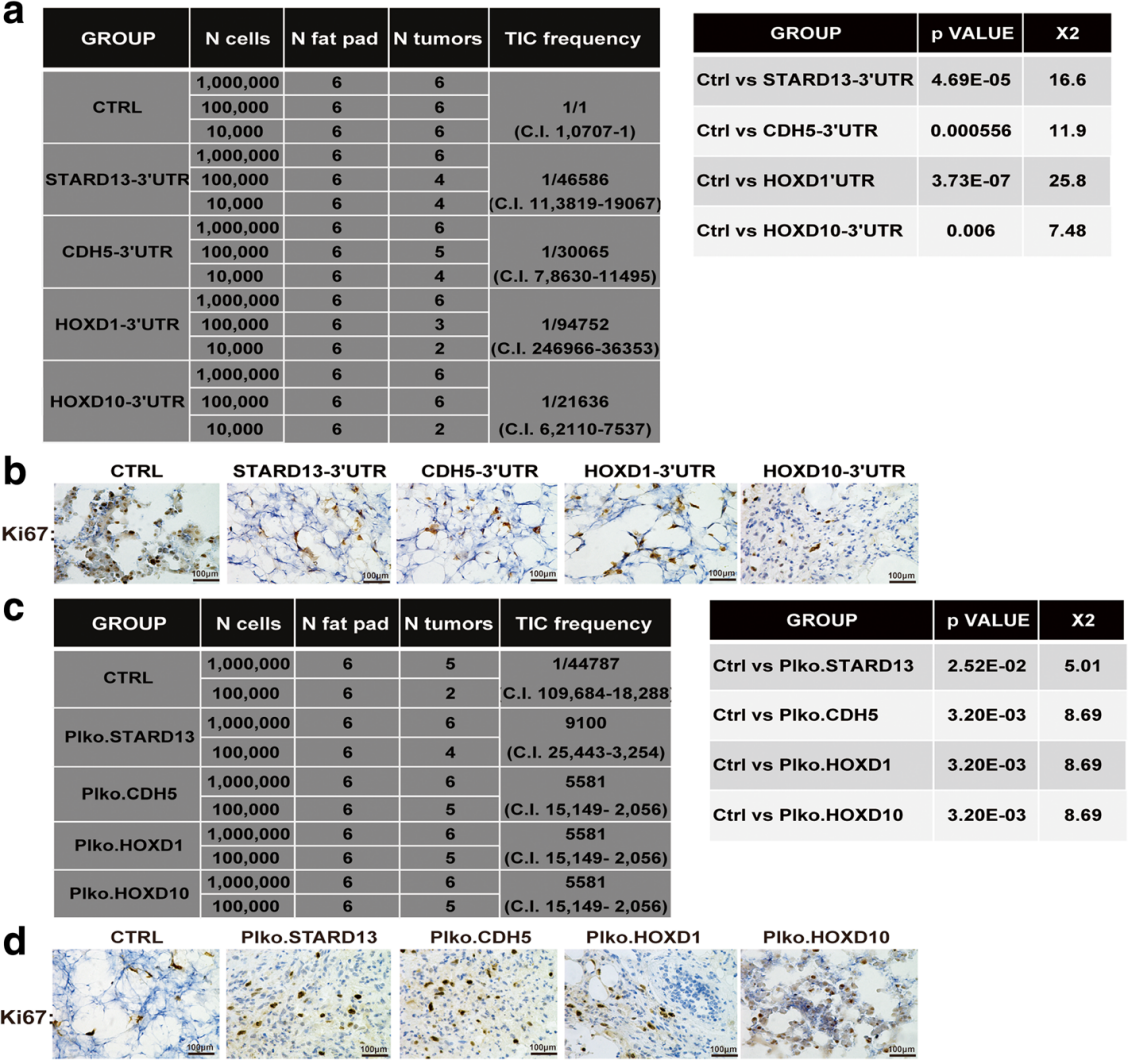
STARD13-correlated ceRNA network inhibits CSC traits of breast cancer cells in vivo. **a** TIC (tumor-initiating cell) frequencies (left), *χ*^2^ values, and associated probabilities (right) of MDA-MB-231 with STARD13-correlated ceRNAs-3′UTR overexpression. Limiting dilution analysis was performed using ELDA software. **b** Represented images of Ki67 staining of tumors harvested when 1,000,000 cells were injected in **a**. **c** TIC frequencies (left), *χ*^2^ values, and associated probabilities (right) of MCF-7 derivatives stably depleted of STARD13 and its ceRNAs (hereafter indicated as pLKO.Ctrl, pLKO.STARD13, pLKO.CDH5, pLKO.HOXD1, and pLKO.HOXD10). Limiting dilution analysis was performed using ELDA software. **d** Represented images of Ki67 staining of tumors harvested when 1,000,000 cells were injected in **c**

We further evaluated whether STARD13-correlated ceRNA network regulates tumor-initiating potential of breast cancer cells in vivo. We compared the capacity of STARD13-correlated ceRNAs-3′UTR-overexpressed MDA-MB-231 cells to seed tumors at limiting dilutions. Although all cell lines could form tumors at a density of 1 × 10^6^ cells, STARD13-correlated ceRNAs-3′UTR-overexpressed cells showed a decrease of tumor size and weight (Additional file 7: Figure S3A and Fig. 2a). Notably, the tumor-seeding ability of STARD13-correlated ceRNAs-3′UTR-overexpressed cells was significantly decreased at a density of 1 × 10^5^ and 1 × 10^4^ cells (Additional file 7: Figure S3B and S3C and Fig. 2a). Concordantly, the staining intensity and number of Ki67, which is required for the maintenance of CSCs [29], were decreased in tumors derived from STARD13-correlated ceRNAs-3′UTR-overexpressed cells (Fig. 2b). Additionally, we further performed in vivo tumorigenic assay with MCF-7 cells after STARD13-correlated ceRNA knockdown and indicated that the knockdown of STARD13-correlated ceRNAs held much stronger tumor-initiating potentials as compared with control cells (Additional file 7: Figure S3D and S3E and Fig. 2c) and increased staining intensity and number of Ki67 (Fig. 2d). Taken together, these results indicate that STARD13-correlated ceRNA network inhibits the tumor initiation ability of breast cancer.

### STARD13-correlated ceRNA network activates Hippo signaling

**Fig. 3:**
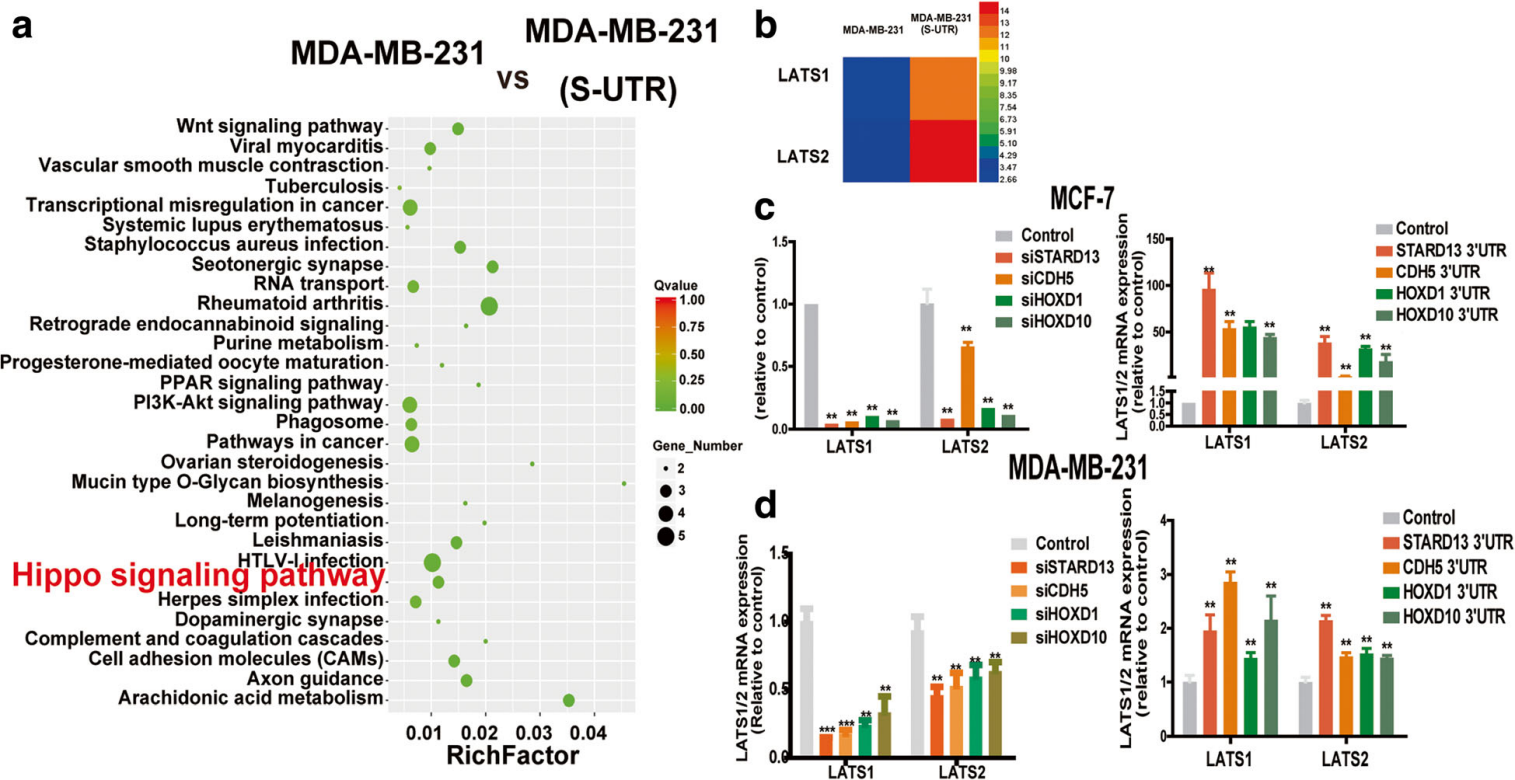
STARD13-correlated ceRNA network activates Hippo signaling via increasing LATS1/2 expression [1]. **a**, **b** Enrichment of signaling signatures differentially expressed (**a**) and identification of LATS1/2 expression (**b**) between pLVX-Ctrl and pLVX-STARD13-3′UTR based on RNA-sequencing analysis. **c**, **d** LATS1/2 was downregulated when STARD13-correlated ceRNAs were depleted and upregulated when overexpressing STARD13-correlated ceRNAs-3′UTRs in MCF-7 (**c**) and MDA-MB-231 cells (**d**). Data were presented as the mean ± SD, *n* = 3, **p < 0.01 vs. vector or control

**Fig. 4:**
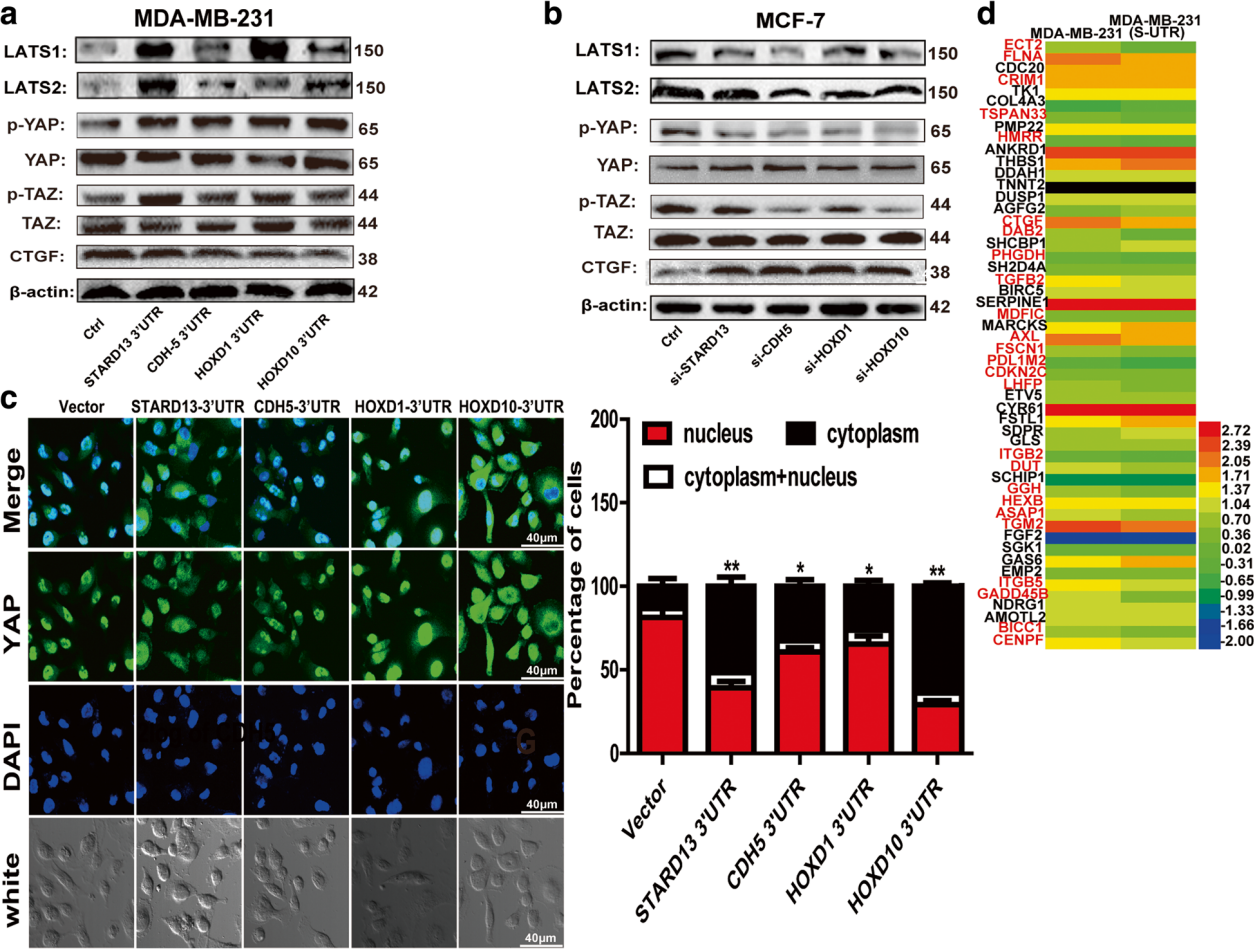
STARD13-correlated ceRNA network activates Hippo signaling via increasing LATS1/2 expression [2]. **a**, **b** Western blot analysis of lysates from MDA-MB-231 cells overexpressing STARD13-correlated ceRNAs-3′UTRs (**a**) and MCF-7 cells depleted of STARD13-correlated ceRNAs (**b**). **c** Confocal images showing localization of YAP in MDA-MB-231 cells overexpressing STARD13-correlated ceRNAs-3′UTRs. **d** Expression of YAP targeting genes in pLVX-Ctrl and pLVX-STARD13-3′UTR cells based on RNA-sequencing analysis. Data were presented as the mean ± SD, *n* = 3, **p < 0.01 vs. vector or control

To explore the related mechanisms contributing to STARD13-correlated ceRNA network in prohibiting breast cancer CSC formation, we tried to characterize the pathways regulated by STARD13-correlated ceRNA network. Firstly, we analyzed the transcriptome of MDA-MB-231 cells with STARD13-3′UTR stable expression or not based on RNA sequencing data. Hippo signaling was shown on the top of the most upregulated pathways by STARD13-3′UTR overexpression (Fig. 3a), and LATS1/2 expression was markedly increased in STARD13-3′UTR-overexpressed cells (Fig. 3b). By analyzing the mRNA microarrays from TCGA, we determined that LATS1/2 level was positively correlated with STARD13, CDH5, HOXD1, and HOXD10 levels in breast cancer tissues, respectively (Additional file 8: Figure S4A). And the ceRNA sequences and the genomic locus were denoted in Additional file 8: Figure S4B. LATS1/2 is the key component of Hippo signaling pathway, consisting of a core kinase cascade of MST1/2, LATS1/2, and downstream effectors YAP/TAZ. Consistently, ectopic expression of STARD13-correlated ceRNAs-3′UTRs in breast cancer cells elevated LATS1/2 levels, while the knockdown of STARD13-correlated ceRNAs decreased LATS1/2 levels (Figs. 3c, d and 4a, b). We also examined the phosphorylation level of YAP/TAZ on Ser-127/Ser-66, which is phosphorylated by LATS1/2 and associated to YAP/TAZ cytoplasmic retention [30], thus being sequestered in the cytoplasm by 14-3-3 proteins [31]. Enforcing the expression of STARD13-correlated ceRNAs-3′UTRs in MDA-MB-231 cells increased the phosphorylation of YAP/TAZ and their cytoplasm retention and dampened the expression of CTGF and the target of YAP (Fig. 4a, c and Additional file 9: Figure S5). Importantly, among 52 YAP-regulated genes examined, 25 genes were commonly reduced when overexpressing STARD13-3′UTR, the core member of STARD13-correlated ceRNA network (Fig. 4d). Conversely, STARD13-correlated ceRNA knockdown resulted in YAP/TAZ dephosphorylation and upregulation of CTGF expression in MCF-7 cells (Fig. 4b). Collectively, our results indicate that STARD13-correlated ceRNA network could activate Hippo signaling.

### STARD13-correlated ceRNA network acts as a sub-ceRNA network to activate Hippo signaling

**Fig. 5:**
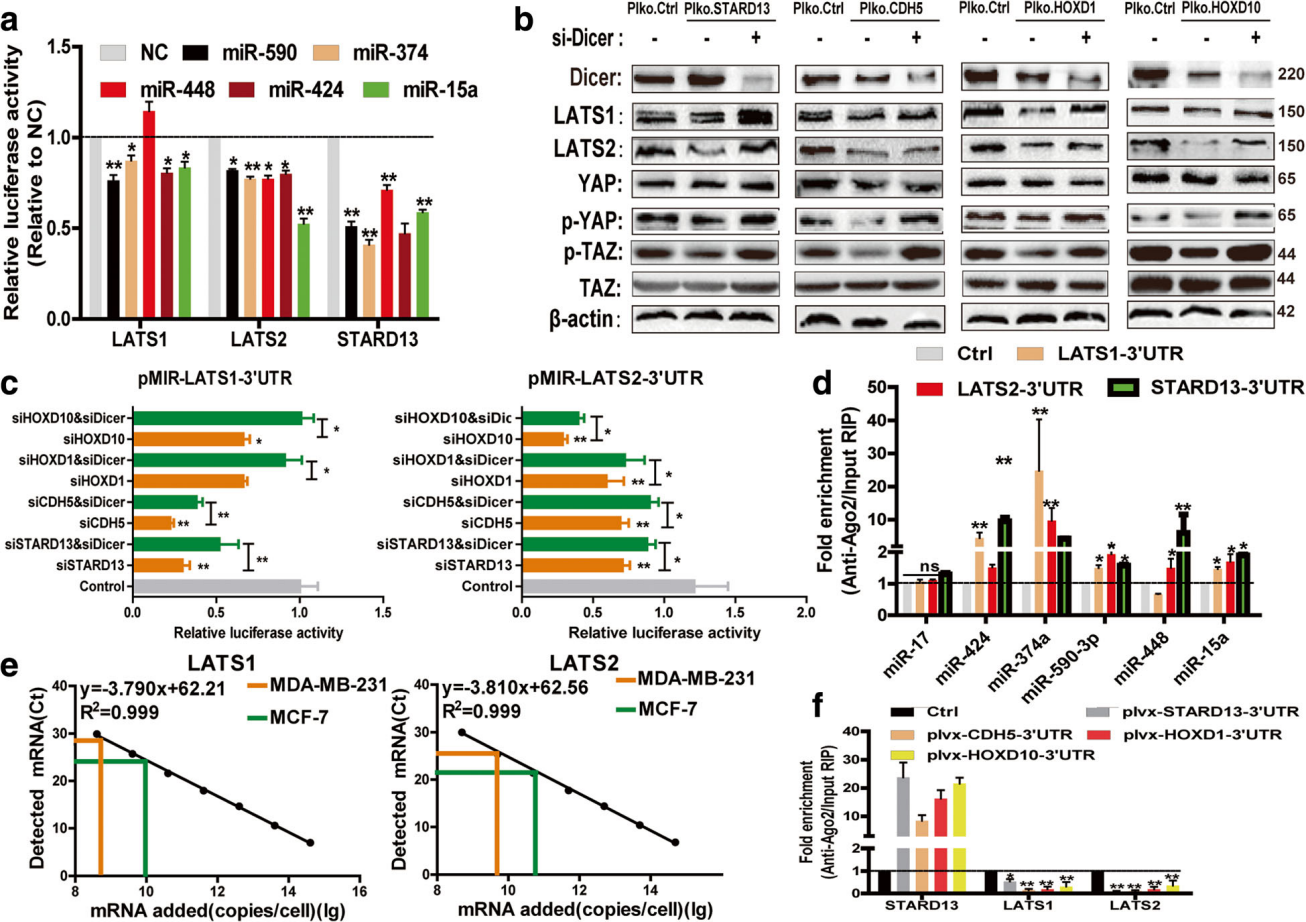
STARD13-correlated ceRNA network acts as a sub-ceRNA network to increase LATS1/2 expression. **a** Luciferase activity of pMIR reporter containing LATS1- or LATS2- or STARD13-3′UTR was detected upon co-transfecting with indicated miRNA mimics in 293T cells. pMIR-reporter empty vector was used as positive control. Data are presented as the ratio of luciferase activity to β-gal activity. **b** Western blot analysis of lysates from MCF-7 cells with STARD13-correlated ceRNA knockdown plus a control or anti-Dicer siRNA co-transfection. Depletion of Dicer rescued the expressions of LATS1/2 and downstream YAP/TAZ phosphorylation in STARD13-correlated ceRNA knocked down MCF-7 cells. **c** Luciferase activity of pMIR reporters which contained LATS1 (left panel) or LATS2 (right panel) 3′UTR with indicated treatment in MCF-7 cells. **d** qRT-PCR was used to measure the level of enrichment of miRNAs in the anti-Ago2-immunoprecipitated complexes in MCF-7 cells with empty vector or pMIR-LATS1-3′UTR or pMIR-LATS2-3′UTR or pLVX-STARD13-3′UTR transfection. miR-17 which did not hold binding sites on LATS1/2 and STARD13 was served as a negative control. **e** Copy number of LATS1 (left) and LATS2 (right) per cell was measured in MCF-7 cells calibrated with an internal standard curve of synthetic constructs containing LATS1 3′UTR or LATS2 3′UTR. **f** RIP assay of enrichment of STARD13, LATS1, and LATS2 transcripts on Ago2 relative to input in MDA-MB-231 cells with STARD13-correlated ceRNAs-3′UTR overexpression. Data were presented as the mean ± SD, *n* = 3, *p < 0.05, **p < 0.01 vs. control/vector

We sought to investigate the mechanisms by which STARD13-correlated ceRNA network regulates Hippo signaling. By computational prediction, LATS1/2-3′UTR was shown to share 16 miRNA binding sites with STARD13-3′UTR (Additional file 10: Table S5). This result was provocative because the previous studies have indicated that the more miRNAs are shared, the more probability of ceRNA network exists [32–34], which led us to examine whether STARD13-3′UTR could activate Hippo signaling by acting as a ceRNA for LATS1/2 and thus regulate LATS1/2 expression. To validate this hypothesis, we examined several predicted shared miRNAs using 3′UTR-luciferase reporter assays. Five miRNAs (miR-424, miR-374a, miR-590-3p, miR-448, and miR-15a) significantly repressed the luciferase activity of STARD13- or LATS1/2-3′UTR-luciferase reporters (except for miR-448 on LATS1) (Fig. 5a), suggesting that the crosstalk between STARD13 and LATS1/2 may be at least in part by these five miRNAs. In the light of the crucial role of miRNAs in ceRNA networks, we sought to knockdown Dicer, an important enzyme for miRNA biogenesis [15]. The knockdown of STARD13-correlated ceRNAs failed to regulate LATS1/2 protein level when Dicer was knocked down (Fig. 5b). Notably, YAP/TAZ dephosphorylation induced by STARD13-correlated ceRNA knockdown was abolished by knocking down Dicer enzyme in MCF-7 cells (Fig. 5b), suggesting that the regulation is miRNA dependent. To further validate that STARD13-correlated ceRNAs share miRNA-binding sites at 3′UTR of LATS1/2 transcripts, we conducted luciferase reporter assays to examine whether STARD13-correlated ceRNAs could compete for the miRNAs targeting at LATS1/2 3′UTR. The knockdown of STARD13-correlated ceRNAs reduced the luciferase activity of pMIR-LATS1/2-3′UTR; this effect was attenuated by knocking down Dicer enzyme (Fig. 5c). Those data further supported the idea that LATS1/2 could be regulated by STARD13-correlated ceRNA 3′UTRs through competing for shared miRNAs. To further confirm the direct interaction between these five miRNAs and STARD13 or LATS1/2 at endogenous levels, we performed RIP analysis to pull down endogenous miRNAs associated with Ago2 in STARD13- and LATS1/2-3′UTR-overexpressed cells. The precipitated miRNAs were subjected to qRT-PCR analysis, and the results showed that these five miRNAs were enriched in RNAs retrieved from STARD13- and LATS1/2-3′UTR-overexpressed cells (Fig. 5d), supporting that STARD13 and LATS1/2 were the bona fide targets of these five miRNAs. The qRT-PCR analysis combined with an internal standard curve revealed that LATS1 and LATS2 were expressed at 8.82 × 10^9^ and 3.02 × 10^10^ in MCF-7 cells and 6.02 × 10^8^ and 3.34 × 10^9^ in MDA-MB-231 cells, respectively (Fig. 5e). As our previous study has shown that STARD13 was expressed at 1.22 × 10^10^ and 8.06 × 10^8^ in MCF-7 and MDA-MB-231 cells, respectively [15]. These results are consistent with the previous reports that ceRNA interaction is optimal when the transcript abundance of ceRNAs within a network is near equimolarity [33, 35, 36]. Next, to assess directly whether STARD13-3′UTR induces LATS1/2 expression through competing for miRNAs, we performed RIP assays against Ago2 to address whether enforced expression of STARD13-3′UTR could displace LATS1/2 transcripts away from RISC complex. Overexpression of STARD13-correlated ceRNAs-3′UTRs resulted in a substantial decrease of LATS1/2 recruitment to Ago2 (Fig. 5f). Notably, the above five miRNA mimics (miR-424, miR-374a, miR-590-3p,miR-448, and miR-15a) were transfected into MDA-MB-231 cells with STARD13-correlated ceRNAs-3′UTR overexpression, and we found that the overexpression of these target miRNAs abolished the effects of STARD13-correlated ceRNA network on LATS1/2 and its downstream effectors p-YAP/p-TAZ expression (Additional file 11: Figure S6). Thus, these results suggest that STARD13-correlated ceRNAs release LATS1/2 transcripts from the post-transcriptional repression mediated by RISC complexes.

Furthermore, we sought to confirm the central role of STARD13 in this ceRNA network, namely, STARD13 ceRNAs (CDH5, HOXD1, and HOXD10)-3′UTRs regulate LATS1/2 expression through STARD13. As expected, the upregulation of LATS1/2 and p-YAP/TAZ levels and downregulation of YAP target and CTGF expression level, both were induced by STARD13 ceRNAs-3′UTR overexpression, were attenuated by STARD13 knockdown, while the overexpression of STARD13-3′UTR decreased or even reversed the knockdown of STARD13 ceRNA-caused downregulation of LATS1/2 and p-YAP/TAZ levels and upregulation of CTGF level (Additional file 12: Figure S7A and S7B). Consistently, the impaired mammary sphere formation capacity resulted from CDH5-, HOXD1-, and HOXD10-3′UTR overexpression was attenuated or reversed by STARD13 knockdown (Additional file 12: Figure S7C). Additionally, the shrinking of CD44+/CD24− subpopulation caused by CDH5-, HOXD1-, and HOXD10-3′UTR overexpression was attenuated or reversed after STARD13 depletion (Additional file 12: Figure S7D). We further tested whether this interaction occurred in vivo as well. Expectedly, the protein levels of LATS1/2 were remarkably upregulated in the tumors derived from the STARD13-correlated ceRNAs-3′UTR-overexpressing cells (Additional file 12: Figure S7E) via immunohistofluorescence stain assay, and the overexpression of STARD13-correlated ceRNAs-3′UTRs elevated the phosphorylation level of YAP through immunohistochemistry analysis (Additional file 12: Figure S7E). All those data testify our hypothesis that STARD13-correlated ceRNA network regulates LATS1/2 expression through acting as a sub-ceRNA network for LATS1/2.

### STARD13-correlated ceRNA network regulates breast CSC traits and EMT through LATS1/2

**Fig. 6:**
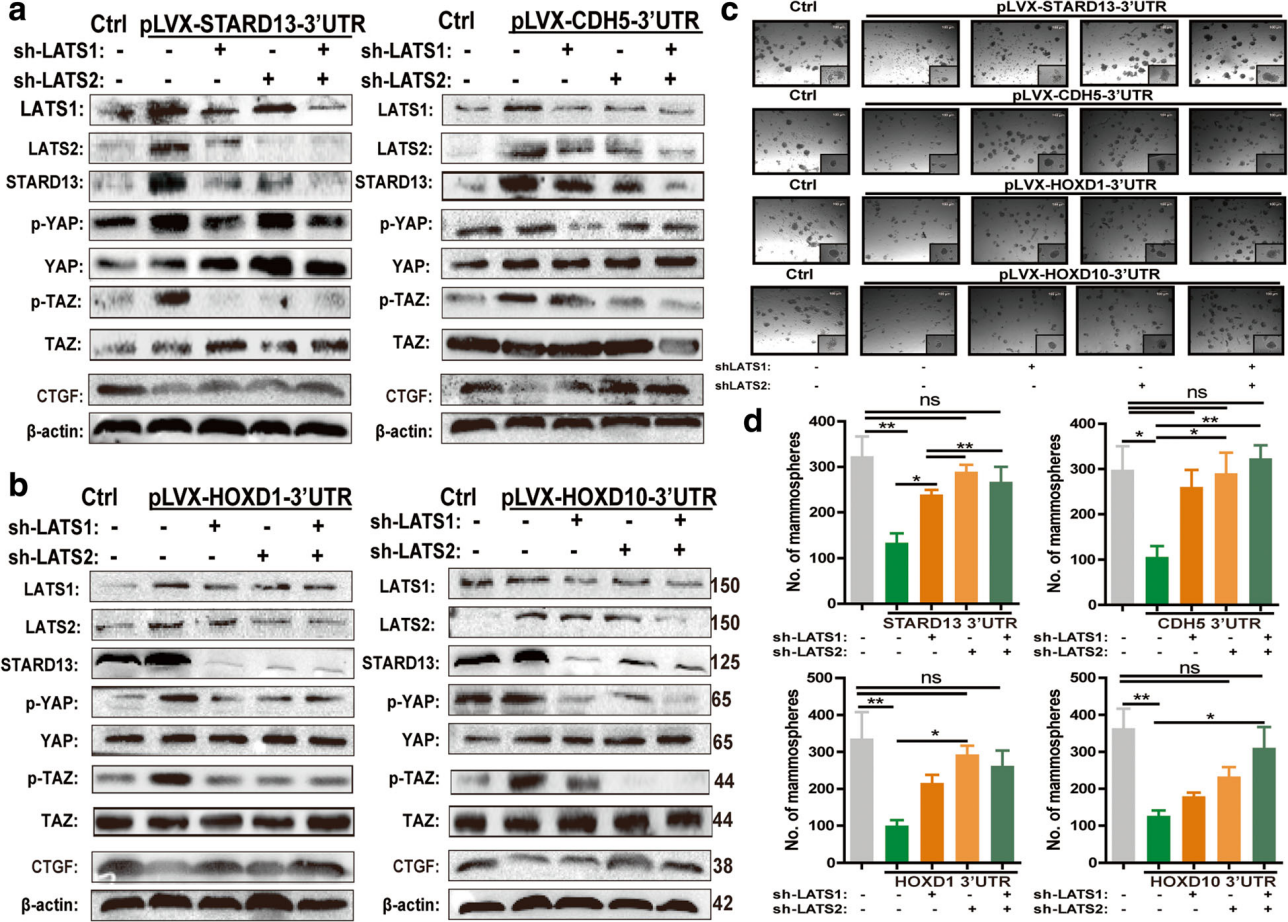
STARD13-correlated ceRNA network regulates breast CSC traits through LATS1/2. **a** and **b** Western blot analysis of lysates from MDA-MB-231 with STARD13- or its ceRNAs-3′UTR overexpression plus LATS1 or LATS2 or LATS1/2 knockdown by lentiviral infection. **c** Phase contract images of mammospheres formed by MDA-MB-231 cells described in **a**. Depletion of LATS1 or LATS2 or LATS1/2 rescued the mammosphere formation impaired by enforced STARD13-correlated ceRNAs-3′UTR expression in MDA-MB-231 cells. **d** Quantification of mammosphere formation in **c**. Data were presented as the mean ± SD, *n* = 3, *p < 0.05, **p < 0.01 vs. pLVX-Ctrl

We then asked whether STARD13-correlated ceRNA network modulates breast cancer cells stemness through LATS1/2. LATS1/2 was knocked down in MDA-MB-231 cells with STARD13-correlated ceRNAs-3′UTR overexpressing by lentivirus shRNAs infection (Fig. 6a, b). Additionally, both the upregulation of p-YAP/TAZ level and the downregulation of its target (CTGF) induced by STARD13-correlated ceRNAs-3′UTR overexpression were attenuated by LATS1/2 knockdown (Fig. 6a, b). LATS1/2 knockdown attenuated the shrinking of the mammary sphere formation and CD44+/CD24− subpopulation, which were both resulted from STARD13-correlated ceRNAs-3′UTR overexpression (Fig. 6c, d and Additional file 13: Figure S8). Our previous study has shown that STARD13-correlated ceRNA network could inhibit breast cancer metastasis by suppressing EMT process [15]. It led us to explore that whether the inhibition of EMT caused by STARD13-correlated ceRNA network is mediated through its effects on LATS1/2 expression. Overexpression of STARD13-correlated ceRNAs impaired mesenchymal marker expression such as N-cadherin, MMP-9, α-SMA, and vimentin, and these effects were attenuated or reversed by knocking down LATS1 or LATS2 separately or simultaneously (Additional file 14: Figure S9). These data suggest that STARD13-correlated ceRNA network suppresses breast cancer EMT and CSC traits at least partly through Hippo signaling pathway.

### STARD13-correlated ceRNA network regulates YAP nuclear abundance by inhibiting RhoA-ROCK signaling

**Fig. 7:**
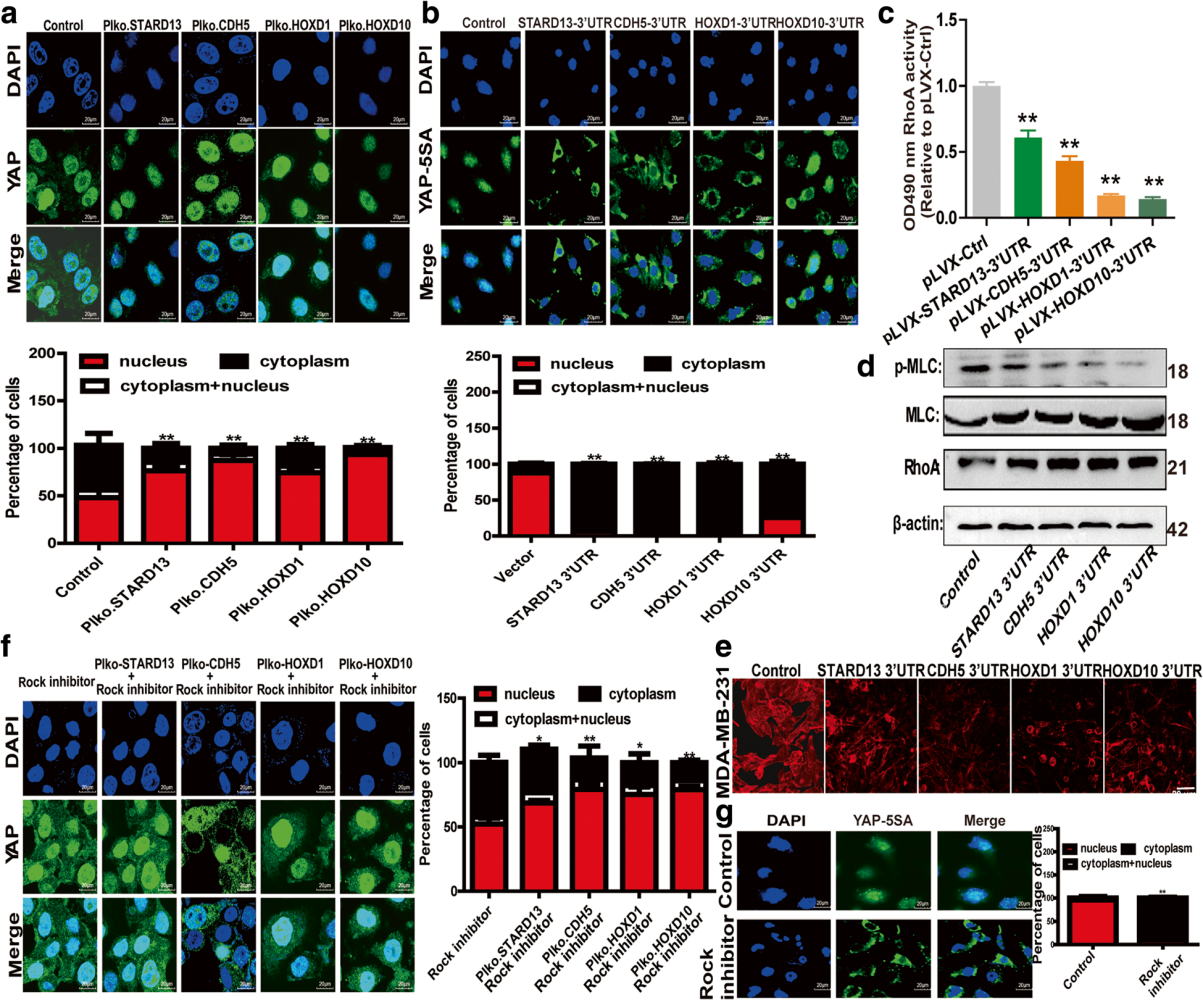
STARD13-correlated ceRNA network regulates YAP nuclear abundance by inhibiting RhoA-ROCK signaling. **a** Confocal images of YAP distribution in MCF-7 cells with STARD13-correlated ceRNAs knockdown. **b** Confocal images of YAP-5SA distribution in MDA-MB-231 cells with STARD13-correlated ceRNAs-3′UTR overexpression or not. Overexpression of STARD13-correlated ceRNAs-3′UTRs could promote YAP-5SA cytoplasmic retention even when it could not be phosphorylated by LATS1/2. **c** Glisa assay of MDA-MB-231 cells with STARD13-correlated ceRNAs-3′UTR overexpression. Overexpression of STARD13-correlated ceRNAs-3′UTRs impaired RhoA activity in MDA-MB-231 cells. **d** Western blot assay of lysates from MDA-MB-231 cells described in **c**. Overexpression of STARD13-correlated ceRNAs-3′UTRs in MDA-MB-231 cells dephosphorylated MLC. **e** Confocal images of filamentous actin (F-actin) stained with rhodamine-labeled phalloidin in MDA-MB-231 cells described in **c**. **f** Confocal images of YAP distribution in MCF-7 cells with STARD13-correlated ceRNA knockdown plus ROCK inhibitor (Y-27632) treatment or not. **g** Confocal images of YAP-5SA distribution in MDA-MB-231 cells with ROCK inhibitor (Y-27632) treatment or not. Data were presented as the mean ± SD, *n* = 3, **p < 0.01 vs. pLVX-Ctrl

As the previous studies have shown that knockdown of LATS had marginal effects on YAP/TAZ signaling in dense cultures [21, 25] which seems to contradict with our results that knockdown of STARD13-correlated ceRNAs could rescue YAP/TAZ nuclear localization in dense monolayers (Fig. 7a and Additional file 15: Figure S10A). Importantly, a mutant form of YAP (YAP-5SA) was transiently transfected in MDA-MB-231 cells to escape LATS1/2-mediated phosphorylation. YAP-5SA was still sensitive to with STARD13-correlated ceRNAs-3′UTR overexpression (Fig. 7b), suggesting that STARD13-correlated ceRNA network could regulate YAP subcellular localization in a LATS1/2-independent manner. These results promote us to explore whether STARD13-correlated ceRNA network regulates YAP nuclear localization via another signaling pathway independent of LATS1/2. STARD13 was demonstrated by our groups and others to block RhoA-ROCK signaling axis by acting as a Rho GTPase-activating proteins (GAP), thus disorganizing F-actin structures [37]. Moreover, the fact that LATS and F-actin organization act independently to regulate YAP/TAZ is also supported by genetic evidence in *Drosophila* [25]. Thus, we conclude that STARD13-correlated ceRNA network might also regulate YAP/TAZ activity through RhoA-ROCK pathway. As expected, Glisa assay indicated that enforcing STARD13-correlated ceRNAs-3′UTR expressions in MDA-MB-231 cells reduced Rho-GTPase activity (Fig. 7c). In line with this, MLC phosphorylation was dampened, but RhoA expression was unaffected (Fig. 7d), and impressive disorganization of F-actin occurred in STARD13- or its ceRNAs-3′UTR-overexpressed MDA-MB-231 cells (Fig. 7e). Notably, cytoplasm abundance of YAP/TAZ was recovered by ROCK inhibitor in MCF-7 cells (Fig. 7f and Additional file 15: Figure S10B), and ROCK inhibitor could also facilitate the cytoplasmic retention of YAP-5SA in MDA-MB-231 cells (Fig. 7g), indicating STARD13-correlated ceRNA network regulates YAP/TAZ nuclear translocation at least partly through RhoA/ROCK/F-actin axis. Moreover, ROCK inhibitor decreased the CD44+/CD24− subpopulation and the promotion of STARD13 knockdown on CD44+/CD24− subpopulation (Additional file 15: Figure S10C). In conclusion, our data provide evidence that STARD13-correlated ceRNA network could regulate YAP/TAZ nuclear abundance in a LATS-independent manner, but through disorganizing F-actin formation by inhibiting RhoA-ROCK signaling.

### STARD13-correlated ceRNA network sensitizes breast cancer cells to doxorubicin

**Fig. 8:**
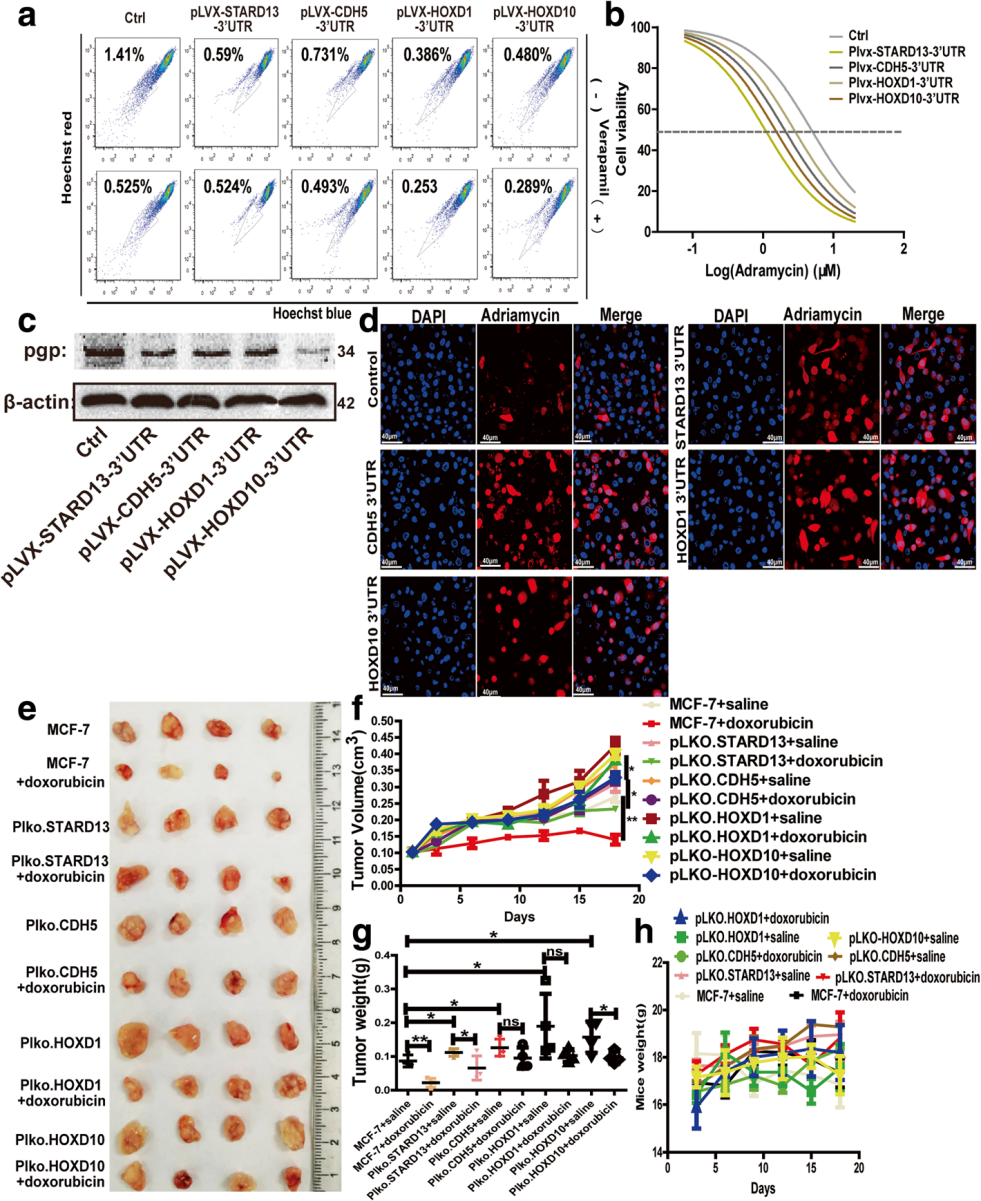
STARD13-correlated ceRNA network enhances the sensitivity of breast cancer cells to doxorubicin. **a** Representative profile of side population (SP) fractions of MDA-MB-231 cells with STARD13-correlated ceRNAs-3′UTR overexpression stained with Hoesch 33342. **b** IC_50_ curves of MDA-MB-231 cells with STARD13-correlated ceRNAs-3′UTR overexpression and were fitted with a nonlinear regression model and were presented as log (doxorubicin) vs. cell viability. **c** Pgp protein expression was detected in cells described in **a**. **d** Confocal images of MDA-MB-231 cells described in **a** with doxorubicin treatment. Overexpression of STARD13-correlated ceRNAs-3′UTRs promoted the cellular retention of doxorubicin. **e** Images of tumors harvested when STARD13-correlated ceRNA stable knockdown cells were planted and followed by doxorubicin treatment or not. **f** The volume of tumors harvested in **e** was monitored. **g** The weight of tumors harvested in **e** was monitored. **h** The weight of mice depicted in **e** was monitored. Data were presented as the mean ± SD, *n* = 3, **p < 0.01 vs. pLVX-Ctrl

We have established the inhibitory role of STARD13-correlated ceRNA network in conferring CSC traits to breast cancer cells. Conferring CSC traits was well confirmed to endow chemoresistance to tumor cells [4]. It led us to speculate whether STARD13-correlated ceRNA network could also sensitize breast cancer cells to doxorubicin. Therefore, we sought to examine the doxorubicin response in MDA-MB-231 cells with STARD13- or ceRNAs-3′UTR overexpression. We noticed that the overexpression of STARD13- or its ceRNAs-3′UTR decreased the activity of multidrug resistance (MDR) proteins, as revealed by the enlargement of SP (Fig. 8a), and enhanced the cell sensitivity to doxorubicin characterized as the decrease of IC_50_ value (Fig. 8b). Additionally, Pgp, an MDR protein, was also downregulated in MDA-MB-231 cells with STARD13- or ceRNAs-3′UTR overexpression (Fig. 8c). Notably, the overexpression of STARD13- or ceRNAs-3′UTR enhanced the intake of doxorubicin in MDA-MB-231 cells (Fig. 8d). On the contrary, STARD13 or its ceRNA knockdown decreased the cell sensitivity to doxorubicin characterized as the increase of IC_50_ value (Additional file 16: Figure S11A) and upregulated Pgp expression levels (Additional file 16: Figure S11B) in MCF-7 cells. Importantly, STARD13 or its ceRNA knockdown decreased doxorubicin sensitivity (Fig. 8e–h), while the overexpression of STARD13 3′UTR enhanced doxorubicin sensitivity in vivo (Additional file 16: Figure S11C–E). Consistently, the intake of doxorubicin in MCF-7 cells was dampened under the absence of STARD13-correlated ceRNAs (Additional file 16: Figure S11G). However, the increase of Pgp expression induced by STARD13 or its ceRNA knockdown was attenuated or even reversed by Dicer knockdown (Additional file 16: Figure S11F). Thus, we confirm that STARD13-correlated ceRNA network could increase the sensitivity of breast cancer cells to doxorubicin in ceRNA-dependent.

**Fig. 9:**
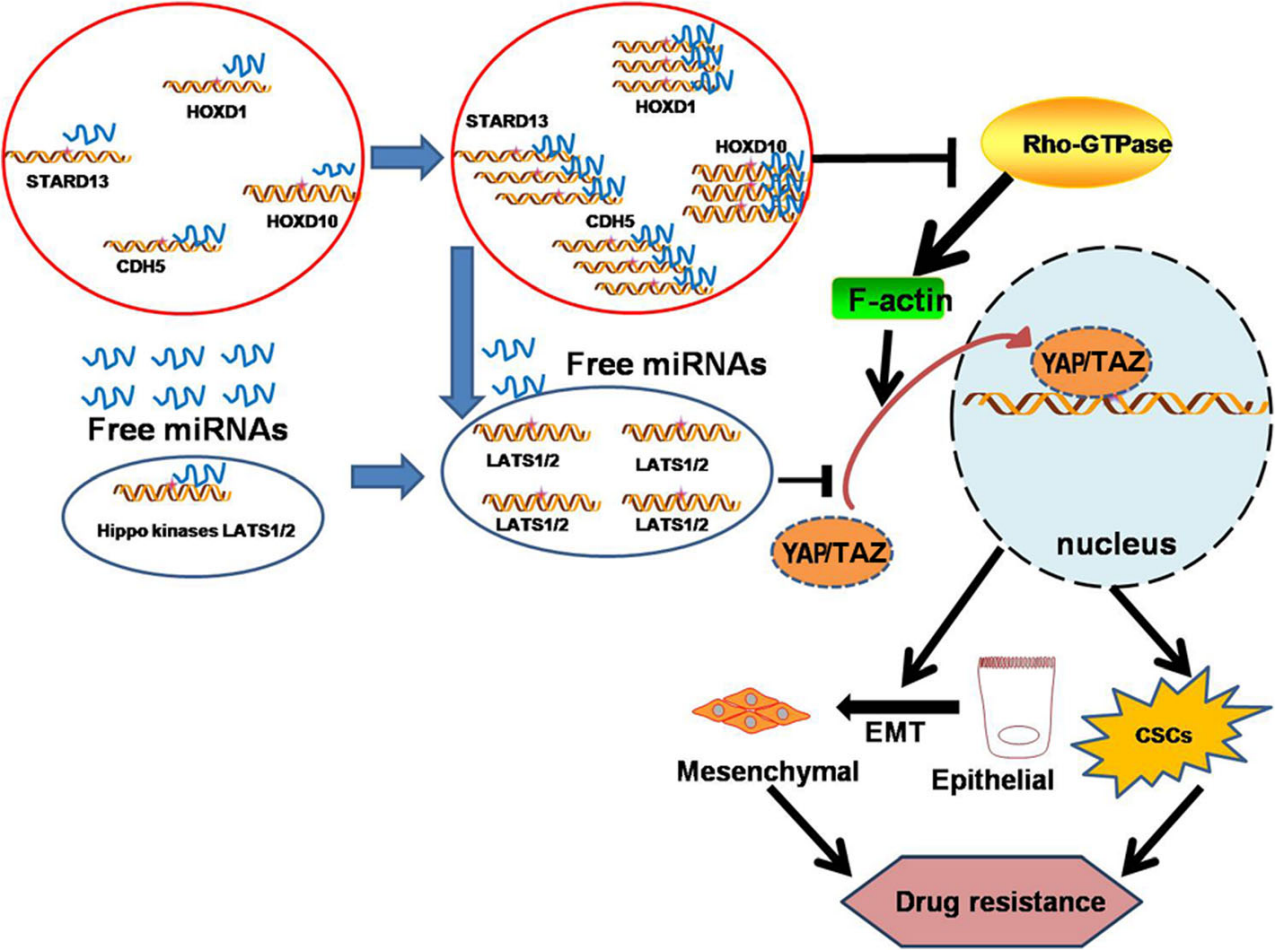
Proposed model that STARD13-correlated ceRNA network inhibits breast cancer EMT and CSC formation, and thus drug resistance. STARD13-correalted ceRNA network acts as a potential inhibitor of YAP transcriptional activity at least through two concurring pathways: one is by facilitating LATS1/2 expression via ceRNA interaction and the other is by blocking RhoA-F-actin signaling cascade through Rho GTPase activity of STARD13. This inhibition results in EMT prohibition and inhibits breast CSC formation, and thus enhances drug sensitivity

To sum up, our results demonstrate that STARD13-correlated ceRNA network acts as a potential inhibitor of YAP/TAZ transcriptional activity at least through two concurring pathways: one was by facilitating LATS1/2 expression and the other by blocking RhoA-F-actin signaling cascade. This inhibition results in EMT prohibition and thus inhibits breast CSC formation, and then enhances doxorubicin sensitivity (Fig. 9).

## Discussion

### STARD13-correlated ceRNA network is a molecular determinant of CSC traits in breast cancer

Here, we revealed that STARD13-correlated ceRNA network attenuated breast CSC formation. This conclusion is supported by the following evidence: (1) the mRNA levels of STARD13-correlated ceRNAs are significantly downregulated in both breast CSC subpopulation sorted by cell surface stemness markers and breast cancer spheres; (2) STARD13-correlated ceRNA network inhibits CSC traits of breast cancer in vitro and in vivo; and (3) our previous study has shown that STARD13-correlated ceRNA network activity is a clinically relevant tool to predict the proclivity to develop metastasis and EMT, itself another hallmark of CSC activity [15].

Functionally, we indicated that activating STRAD13-correlated ceRNA network inhibited CSC-related traits, i.e., tumor sphere formation and tumor initiation properties of breast cancer cells while depleting STARD13 or its ceRNAs promoted these CSC-related traits. Thus, the activity of STARD13-correlated ceRNA network has been linked to breast CSCs, i.e., tumor seeding. Interestingly, the previous study has shown that normal and non-stem cells could spontaneously revert to a stem-like state under appropriate intrinsic or extrinsic cues [5]. The unsettled issue is whether the effects of STARD13-correlated ceRNA network on breast CSCs also exist in non-stem cells, such as mammary epithelial cells.

### A mechanism that links STARD13-correlated ceRNA network to Hippo signaling

In addition, we have revealed the STARD13-correlated ceRNA network as a deterministic upstream controller of YAP/TAZ transcriptional activity. Recent studies have uncovered ceRNA network as a crucial modulator in human cancer [38, 39]. Characterizing the identity, function, and mechanism of the ceRNAs will not only further our fundamental understanding of RNA-mediated cancer pathogenesis but may also shed light on the development of new RNA-based therapeutic strategies for treating cancer [13]. Notably, extensive ceRNA-ceRNA interaction networks could regulate development in multiple rhesus tissues [39]. Such indirect interactions critically result in ceRNA crosstalk in a larger ceRNA network, and sub-networks contained within larger networks could explain the pronounced crosstalk alterations observed when distal potent ceRNAs are silenced [40], such as we have previously demonstrated that the ceRNA network between pseudogene CYP4Z2P and CYP4Z1 could enhance ERα transcriptional activity, thus conferring tamoxifen resistance in MCF-7 cells via acting as a sub-network for CDK3 in ER-positive breast cancer [41]. We further showed that this ceRNA network exerts an anti-apoptotic function via acting as a sub-network for hTERT in breast cancer cells [42]. In this work, we showed that STARD13-correlated ceRNA network acted as a sub-network for LATS1/2 and thus promoted LATS1/2 activity, demonstrating a role of this ceRNA network in modulating Hippo pathway. These molecular and cellular findings are corroborated by the results that CDH5-, HOXD1-, and HOXD10-3′UTR could regulate LATS1/2 activity and downstream Hippo signaling in an miRNA-dependent manner. However, the luciferase reporter assay showed that miR-448 did not decrease the luciferase activity of LATS1 (Fig. 5a), which is in accordance with the result of RIP assay with Ago2 (Fig. 5d). These results indicate that the ceRNA interaction between STARD13-correlated ceRNA network and LATS1 is at least through the other four miRNAs. Of note, CDH5-, HOXD1-, and HOXD10-3′UTRs could not work in this way without STARD13, suggesting a key and bridge role of STARD13 in this larger ceRNA network, which communicated its ceRNAs (CDH5, HOXD1, and HOXD10) with LATS1/2, the crucial member of Hippo signaling cascade. In line with this, the absence of LATS1/2 attenuated the inhibitory roles of STARD13-correlated ceRNA network on CSC and EMT traits, highlighting that STARD13-correlated ceRNA network exerts its tumor-suppressive effects at least partly through regulating LATS1/2. Notably, genetically depleting LATS1/2 in MCF-7 cells did not additionally attenuate the inhibitory effects of STARD13-correlated ceRNA network compared to depleting LATS1 or LATS2 alone, demonstrating that alternative activation of LATS1 or LATS2 is sufficient to inhibit YAP activity and downstream effects.

### A mechanism that links STARD13-correlated ceRNA network to Rho-GTPase/F-actin signaling

Data from multiple groups, including our own, have shown that STARD13 could serve as a Rho GTPase-activating protein (GAP) to inhibit Rho-GTPases and thus RhoA activity, resulting in the reorganization of the cytoskeleton [37, 43]. Previous studies have indicated that Rho-GTPase and F-actin rearrangements are required for YAP/TAZ activity [19, 23]. Galphaq stimulates YAP/TAZ activity through a Trio-Rho/Rac signaling circuitry promoting actin polymerization, independently of the canonical Hippo pathway [44]. Additionally, the regulators of Rho-GTPase could modulate cell shape and YAP/TAZ localization in triple negative breast cancer [45]. YAP/TAZ responds to mechanical signals from the surrounding ECM, which informs cells the need to preserve stem cell attributes depending on tissue needs, and mechanotransduction is intimately interwoven with cytoskeletal dynamics [22]. Therefore, we speculated that STARD13-mediated inhibition of Rho-GTPase activity could also regulate YAP/TAZ activity. Since STARD13-correlated ceRNAs-3′UTRs could promote STARD13 expression via their ceRNA activities, we strongly believe that STARD13-correlated ceRNA network could also regulate YAP/TAZ activity via inhibiting Rho-GTPase/F-actin signaling. Consistently, Glisa assay confirmed that STARD13-correlated ceRNA network reduced Rho-GTPase activity, and impressive disorganization of F-actin occurred in STARD13- or its ceRNAs-3′UTR-overexpressed MDA-MB-231 cells; this result is also consistent with our recent work [26]. Importantly, cytoplasm abundance of YAP/TAZ and YAP-S5A was recovered by activating STARD13-correlated ceRNA network or Rock inhibitor. These results substantiate our speculation that STARD13-correlated ceRNA network regulates YAP/TAZ activity at least partly through Rho-GTPase/F-actin signaling and beyond through Hippo pathway.

It is worth noting that most studies to date have pointed to YAP/TAZ in the regulation of the Hippo pathway or Rho-GTPase/F-actin pathway alone. Among the numerous upstream signals, mechanical inputs remain a central pillar in the control of YAP/TAZ activity [25] and represent a prerequisite for it. And this is testified by the fact that the loss of LATS1/2 fails to revive YAP/TAZ activity in the cells that are mechanically inhibited [25, 46]. By identifying LATS1/2 and Rho-GTPase as two synergistic instruments of STARD13-correlated ceRNA network in regulating breast CSC traits, our findings forge a novel and unanticipated collaboration between oncogenic Rho-GTPase/F-actin function and the tumor-suppressive Hippo pathway in breast cancer via ceRNA crosstalking.

To sum up, since STARD13-correlated ceRNA network could simultaneously promote LATS1/2 (the suppressor of YAP/TAZ transcriptional activity) expression, and inhibit Rho-GTPase/F-actin signaling cascade (the derepressor of YAP/TAZ nuclear translocation), it might exert a more profound regulation on YAP/TAZ activity than either of those factors alone. Accordingly, one may propose a therapeutic strategy driving STARD13-correlated ceRNA network considering its comprehensive effects on YAP transcriptional activity.

### STARD13-correlated ceRNA network modulates doxorubicin sensitivity via regulating YAP/TAZ activity

Increasing evidence has demonstrated that CSCs play vital roles in breast cancer metastasis, relapse, and chemoresistance. Although traditional therapies work in the early treatment of breast cancers, they fail to target and wipe out the CSCs, which contribute to chemoresistance and tumor recurrence [47]. Notably, salinomycin has been identified to specifically target breast CSCs and thus effectively prohibit breast cancer progression [48]. Additionally, mifepristone, a drug regularly used for abortion, has been shown to inhibit triple negative breast cancer growth via reducing breast CSC population [28]. Therefore, it is an urgent need to develop more therapies specifically targeting CSCs for breast cancer treatment, especially for triple negative breast cancer which innately holds a poorly differentiated “stem/progenitor” cell phenotype [49]. Attributing responses to CSCs is also recommended as the key to accelerating an understanding of their biology and developing more effective methods for their eradication in patients [49].

In this work, we showed that STARD13-correlated ceRNA network could regulate CSC traits of breast cancer cells through two independent pathways, which collaboratively led to the nucleus-cytoplasm translocation of YAP/TAZ. Despite the more details that need to be elucidated, we proposed that verteporfin, an inhibitor of YAP-TEAD binding, might target breast CSCs and could be used as a combinative treatment with doxorubicin in breast cancer therapy. However, we must admit that other first-line drugs in breast cancer treatment were not examined in this work, which could be performed in our future work.

## Conclusions

Our work reveals an unpredicted layer of YAP regulation and put the activation of STARD13-correlated ceRNA network as a potential novel therapeutic strategy to specifically target breast CSCs.

## Additional files

Additional file 1: **Table S1**. Sequences of siRNA against specific target in this study. (DOC 34 kb)

Additional file 2: **Table S2.** Sequences of primers used for qRT-PCR in this study (DOC 44 kb)

Additional file 3: **Table S3.** Primary antibodies used in this study. (DOC 36 kb)

Additional file 4: **Table S4.** Sequences of primers used for plasmid constructions. (DOC 41 kb)

Additional file 5: **Figure S1.** The infection efficiency of lentivirus. (A) Lentiviral infection efficiency of MDA-MB-231 cells stably expressing STARD13-3′UTR, CDH5-3′UTR, HOXD1-3′UTR, and HOXD10-3′UTR was examined by qRT-PCR. (B) Lentiviral infection efficiency of MCF-7 cells stably depleted of STARD13, CDH5, HOXD1, and HOXD10 was verified by Western blot analysis. Data were presented as the mean ± SD, *n* = 3, ***p < 0.001 vs. Ctrl. (TIF 1373 kb)

Additional file 6: **Figure S2.** MCF-7 cells depleted of STARD13-correlated ceRNAs gain CSC traits. (A) Phase contract images of mammospheres formed by MCF-7 cells with STARD13-correlated ceRNA knockdown. (B) Quantification of mammospheres formed in (A). (C) Represented FACS profile of MCF-7 cells described in (A). (D) Identification of stemness-related genes expression (ALDH1, OCT4, and Nanog) by Western blot analysis in MCF-7 cells described in (A). Data were presented as the mean ± SD, *n* = 3, *p < 0.05, **p < 0.01 vs. pLVX-Ctrl. (TIF 3016 kb)

Additional file 7: **Figure S3.** STARD13-correlated ceRNA network inhibits CSC traits of breast cancer cells in vivo. (A, B, and C) Images (left) and weight (right) of tumors harvested when serially diluted MDA-MB-231 cells with STARD13-correlated ceRNAs-3′UTR overexpression were planted. (D and E) Images (left) and weight (right) of tumors harvested when serially diluted MCF-7 cells with STARD13 or its ceRNA knockdown were planted. (TIF 4230 kb)

Additional file 8: **Figure S4.** (A) Correlation analysis between LATS1/2 and STARD13, CDH5, HOXD1, and HOXD10, based on the microarray data downloaded from the TCGA data portal. (B) The ceRNA sequence and the genomic locus. (TIF 1525 kb)

Additional file 9: **Figure S5.** STARD13-correlated ceRNAs-3′UTRs regulate TAZ nuclear abundance. Confocal images of TAZ distribution in MDA-MB-231 cells with STARD13-correlated ceRNAs-3′UTR overexpression or not. Data were presented as the mean ± SD, *n* = 3, *p < 0.05, **p < 0.01 vs. Vector. (TIF 4364 kb)

Additional file 10: **Table S5.** The number of common miRNA binding sites on STARD13 3′UTR and LATS1/2 3′UTR is predicted using Targetscan 6.2 and microRNA.org. (DOC 33 kb)

Additional file 11: **Figure S6.** Target miRNAs attenuated the promotive effects of STARD13-correlated ceRNA network on Hippo signaling. Target miRNAs (miR-424, miR-374a, miR-590-3p, miR-448, and miR-15a) mimics mix was co-transfected with STARD13-correlated ceRNAs-3′UTR overexpression constructs or not, the protein level of LATS1/2 and downstream effectors (p-YAP/p-TAZ, YAP/TAZ, and CTGF) was examined. (TIF 1437 kb)

Additional file 12: **Figure S7.** CDH5, HOXD1, HOXD10-3′UTRs regulate Hippo signaling and CSC traits of breast cancer cells through STARD13. (A) Western blot analysis of lysates from MCF-7 cells with its ceRNA knockdown plus STARD13 3′UTR co-transfection or not. (B) Western blot analysis of lysates from MDA-MB-231 cells with STARD13 ceRNAs-3′UTR overexpression plus STARD13 knockdown or not. (C and D) Phase contract images of mammospheres (C) formed by MDA-MB-231 cells described in (A). Representative FACS profile (D) of them with CD24− and CD44+ markers by flow cytometry analysis. (E) Represented images of p-YAP, LATS1, and LATS2 staining of tumors harvested when 1,000,000 cells were injected in Fig. 2a. (TIF 5010 kb)

Additional file 13: **Figure S8.** Representative FACS profile of MDA-MB-231 cells with STARD13- or its ceRNAs-3′UTR overexpression plus LATS1 or LATS2 or LATS1/2 knockdown by lentiviral infection. (TIF 1568 kb)

Additional file 14: **Figure S9.** STARD13-correlated ceRNA network regulates breast cancer EMT through LATS1/2. EMT marker (see in main text) expressions were measured in MDA-MB-231 cells with STARD13- or its ceRNAs-3′UTR overexpression plus LATS1 or LATS2 or LATS1/2 knockdown by lentiviral infection. (TIF 2846 kb)

Additional file 15: **Figure S10.** STARD13-correlated ceRNA network regulate TAZ nuclear abundance by inhibiting RhoA-ROCK signaling. (A) Confocal images of TAZ distribution in MCF-7 cells with STARD13-correlated ceRNA knockdown. (B) Confocal images of TAZ distribution in MCF-7 cells with STARD13-correlated ceRNA knockdown plus ROCK inhibitor (Y-27632) treatment or not. (C) Representative FACS profile of MCF-7 cells with STARD13 knockdown plus ROCK inhibitor treatment. Data were presented as the mean ± SD, *n* = 3, *p < 0.05, **p < 0.01 VS. Control or Rock inhibitor. (TIF 5007 kb)

Additional file 16: **Figure S11.** Depletion of STARD13-correlated ceRNAs dampens the response of breast cancer cells to doxorubicin. (A) IC_50_ curves of MCF-7 cells with STARD13-correlated ceRNA knockdown and were fitted with a nonlinear regression model and were presented as log (Doxorubicin) vs cell viability. (B) Western blot assay of lysates from MCF-7 cells with STARD13-correlated ceRNA knockdown. (C) Images of tumors harvested when STARD13 3′UTR stable overexpression cells were planted and followed by doxorubicin treatment or not. The weight of tumors harvested in (C) was monitored. (D) The weight of mice depicted in (C) was monitored. (E) The volume of tumors harvested in (C) was monitored. (G) Confocal images of MCF-7 cells described in (B) with doxorubicin treatment. Depletion of STARD13-correlated ceRNAs impaired the cellular retention of doxorubicin. (F) Western blot assay of lysates from MCF-7 cells with STARD13-correlated ceRNA knockdown plus si-Dicer or not. (TIF 3999 kb)

## Abbreviations

ceRNA: Competing endogenous RNAs
CSCs: Cancer stem cells
ECM: Extracellular matrix
EMT: Epithelial-to-mesenchymal transition
FACS: Fluorescence-activated cell sorting
FBS: Fetal bovine serum
GEO: Gene Expression Omnibus
IHC: Immunohistochemistry
LATS: Large tumor suppressor
MDR: Multidrug resistance
qRT-PCR: Quantitative real-time PCR
RIP: RNA immunoprecipitation
STR: Short tandem repeat
TCGA: The Cancer Genome Atlas
UTR: Untranslated region
