# Supplementary material for: STARD13-correlated ceRNA network-directed inhibition on YAP/TAZ activity suppresses stemness of breast cancer via co-regulating Hippo and Rho-GTPase/F-actin signaling
Source: J Hematol Oncol. 2018 May 30;11:72. doi: 10.1186/s13045-018-0613-5 (PMC5977742; doi:10.1186/s13045-018-0613-5)
Supplement: Supplementary file 1 — Table S1. Sequences of siRNA against specific target in this study. (DOC 34 kb) [file 13045_2018_613_MOESM1_ESM.doc]

**Additional file 1: Table S1. Sequences of siRNA against specific target in this study.**

| Name |  | Sequences |
| --- | --- | --- |
| STARD13 siRNA | Sense (5’-3’) | CACCUUUCCAUCUCCUAAUTT |
|  | Anti-Sense (5’-3’) | AUUAGGAGAUGGAAAAGGUGTT |
| CDH5 siRNA | Sense (5’-3’) | GGAACCAGAUGCACAUUGATT |
|  | Anti-Sense (5’-3’) | UCAAUGUGCAUCUGGUUCCTT |
| HOXD1 siRNA | Sense (5’-3’) | CGAGAUAGCCAACUGCUUGTT |
|  | Anti-Sense (5’-3’) | CAAGCAGUUGGCUAUCUCGTT |
| HOXD10 siRNA | Sense (5’-3’) | CGAAUGAAACUCAAGAAGATT |
|  | Anti-Sense (5’-3’) | UCUUCUUGAGUUUCAUUCGTT |
| LATS1 siRNA | Sense (5’-3’) | GCAGCGUCUACAUCGUAAATT |
|  | Anti-Sense (5’-3’) | UUUACGAUGUAGACGCUGCTT |
| LATS2 siRNA | Sense (5’-3’) | GGACUCACAAUUCCAAAUATT |
|  | Anti-Sense (5’-3’) | UAUUUGGAAUUGUGAGUCCTT |
| Dicer siRNA | Sense (5’-3’) | AAGGCUUACCUUCUCCAGGCUTT |
|  | Anti-Sense (5’-3’) | AGCCUGGGAGAAGGUAAGCCTT |
