# Supplementary material for: STARD13-correlated ceRNA network-directed inhibition on YAP/TAZ activity suppresses stemness of breast cancer via co-regulating Hippo and Rho-GTPase/F-actin signaling
Source: J Hematol Oncol. 2018 May 30;11:72. doi: 10.1186/s13045-018-0613-5 (PMC5977742; doi:10.1186/s13045-018-0613-5)
Supplement: Supplementary file 2 — Table S2. Sequences of primers used for qRT-PCR in this study (DOC 44 kb) [file 13045_2018_613_MOESM2_ESM.doc]

**Additional file 2: Table S2.** Sequences of primers used for qRT-PCR in this study

| Name |  | Sequences |
| --- | --- | --- |
| STARD13 | Forward (5’-3’) | GGACCTTGTAGAACCTCTTTGC |
|  | Reverse (5’-3’) | TCATCTGAGTCGTCACCCTTT |
| CDH5 | Forward (5’-3’) | AAGCGTGAGTCGCAAGAATG |
|  | Reverse (5’-3’) | TCTCCAGGTTTTCGCCAGTG |
| HOXD1 | Forward (5’-3’) | CGGGTCTCACGTCCACTAC |
|  | Reverse (5’-3’) | GATGCGGTCTGGAAAGCAC |
| HOXD10 | Forward (5’-3’) | GACATGGGGACCTATGGAATGC |
|  | Reverse (5’-3’) | CGGATCTGTCCAACTGTCTACT |
| LATS1 | Forward (5’-3’) | CACCACCCTACCCAAAACA |
|  | Reverse (5’-3’) | GAGATAATCCAACCCGCAT |
| LATS2 | Forward (5’-3’) | TGGAGTGTTGGAGTGATTC |
|  | Reverse (5’-3’) | TGTTATTGGGCGAGGTGAG |
| ALDH1 | Forward (5’-3’) | AGCCTTCACAGGATCAACAGA |
|  | Reverse (5’-3’) | GTCGGCATCAGCTAACACAA |
| Nanog | Forward (5’-3’) | GCAGGCAACTCACTTTATCC |
|  | Reverse (5’-3’) | CCCACAAATCACAGGCATAG |
| Oct4 | Forward (5’-3’) | AGCGATCAAGCAGCGACTA |
|  | Reverse (5’-3’) | GGAAAGGGACCGAGGAGTA |
| Sox2 | Forward (5’-3’) | CATCACCCACAGCAAATGAC |
|  | Reverse (5’-3’) | CAAAGCTCCTACCGTACCACT |
| Dicer | Forward (5’-3’) | GTACGACTACCACAAGTACTTC |
|  | Reverse (5’-3’) | ATAGTACACCTGCCAGACTGT |
| CTGF | Forward (5’-3’) | TGGAGATTTTGGGAGTACGG |
|  | Reverse (5’-3’) | GCAGGCTAGAGAAGCAGAGC |
| TGF-β | Forward (5’-3’) | CACGTGGAGCTGTACCAGAA |
|  | Reverse (5’-3’) | GAACCCGTTGATGTCCACTT |
| GAPDH | Forward (5’-3’) | CTTAGTTGCGTTACACCCTTTCTTG |
|  | Reverse (5’-3’) | CTGTCACCTTCACCGTTCCAGTTT |
