# Supplementary material for: STARD13-correlated ceRNA network-directed inhibition on YAP/TAZ activity suppresses stemness of breast cancer via co-regulating Hippo and Rho-GTPase/F-actin signaling
Source: J Hematol Oncol. 2018 May 30;11:72. doi: 10.1186/s13045-018-0613-5 (PMC5977742; doi:10.1186/s13045-018-0613-5)
Supplement: Supplementary file 3 — Table S3. Primary antibodies used in this study. (DOC 36 kb) [file 13045_2018_613_MOESM3_ESM.doc]

**Additional file 3: Table S3. Primary antibodies used in this study.**

| Antigens | Manufacturer | Application |
| --- | --- | --- |
| LATS1 | Santa Cruz | 1:1000 for WB |
| LATS2 | Santa Cruz | 1:1000 for WB |
| YAP | Santa Cruz | 1:1000 for WB |
| TAZ | Santa Cruz | 1:1000 for WB |
| P-YAP | Cell Signalling Technology | 1:1000 for WB |
| P-TAZ | Affinity Biosciences | 1:1000 for WB |
| STARD13 | Abcam | 1:1000 for WB |
| CDH5 | Abcam | 1:1000 for WB |
| HOXD1 | Abcam | 1:1000 for WB |
| HOXD10 | Abcam | 1:1000 for WB |
| N-cadherin | Wanleibio (Shenyang, China) | 1:1000 for WB |
| MMP-9 | Wanleibio | 1:1000 for WB |
| α-SMA | Wanleibio | 1:1000 for WB |
| Vimentin | Wanleibio | 1:1000 for WB |
| β-actin | YIFEIXUE BIO TECH | 1:5000 for WB |
| Dicer | Cell Signalling Technology | 1:1000 for WB |
| Ago2 | Cell Signalling Technology | 1:50 for RIP |
