# Supplementary material for: STARD13-correlated ceRNA network-directed inhibition on YAP/TAZ activity suppresses stemness of breast cancer via co-regulating Hippo and Rho-GTPase/F-actin signaling
Source: J Hematol Oncol. 2018 May 30;11:72. doi: 10.1186/s13045-018-0613-5 (PMC5977742; doi:10.1186/s13045-018-0613-5)
Supplement: Supplementary file 4 — Table S4. Sequences of primers used for plasmid constructions. (DOC 41 kb) [file 13045_2018_613_MOESM4_ESM.doc]

**Additional file 4: Table S4. Sequences of primers used for plasmid constructions.**

| Name |  | Sequences |
| --- | --- | --- |
| pLVX-STARD13-3’UTR | Forward (5’-3’) | GGACTAGTCCCCAGAATGGTACAGCAAAG |
|  | Reverse (5’-3’) | GCTCTAGAGAGGGAGAATCAGAAATACAATCAC |
| pLVX-CDH5-  3’UTR | Forward (5’-3’) | GCTCTAGAGCTGTACTGAGCACTGAACCAC |
|  | Reverse (5’-3’) | CGCGGATCCTCTCTGTTGACTGATGCCACTT |
| pLVX-HOXD1-  3’UTR | Forward (5’-3’) | GCTCTAGAACTGTCTTGTAAGCCACTTGTTTG |
|  | Reverse (5’-3’) | CGCGGATCCGCTACATCAAGGAGACCCTAACT |
| pLVX-HOXD10-3’UTR | Forward (5’-3’) | GCTCTAGAGACTTTGGGGTCATTATGTTCG |
|  | Reverse (5’-3’) | CGCGGATCCGGATGCTCTACAGTTCCAATAAGT |
| pLKO.STARD13 | Sense (5’-3’) | CCGGTCACCTTTCCATCTCCTAATCTCGAGATTAGGAGATGGAAAGGTG TTTTTG |
|  | Anti-Sense (5’-3’) | AATTCAAAAACACCTTTCCATCTCCTAATCTCGAGATTAGGAGATGGAAAGGTG A |
| pLKO.CDH5 | Sense (5’-3’) | CCGGTGGAACCAGATGCACATTGACTCGAGTCAATGTGCATCTGGTTCC TTTTTG |
|  | Anti-Sense (5’-3’) | AATTCAAAAAGGAACCAGATGCACATTGACTCGAG TCAATGTGCATCTGGTTCCA |
| pLKO.HOXD1 | Sense (5’-3’) | CCGGTCGAGATAGCCAACTGCTTGCTCGAGCAAGCAGTTGGCTATCTCG TTTTTG |
|  | Anti-Sense (5’-3’) | AATTCAAAAACGAGATAGCCAACTGCTTGCTCGAG CAAGCAGTTGGCTATCTCGA |
| pLKO.HOXD10 | Sense (5’-3’) | CCGGTCGAATGAAACTCAAGAAGACTCGAGTCTTCTTGAGTTTCATTCG TTTTTG |
|  | Anti-Sense (5’-3’) | AATTCAAAAACGAATGAAACTCAAGAAGACTCGAG TCTTCTTGAGTTTCATTCG A |
| pLKO.LATS1 | Sense (5’-3’) | CCGGGCAGCGTCTACATCGTAAACTCGAGTTTACGATGTAGACGCTGCTTTTTG |
|  | Anti-Sense (5’-3’) | AATTCAAAAAGCAGCGTCTACATCGTAAACTCGAGTTTACGATGTAGACGCTGC |
| pLKO.LATS2 | Sense (5’-3’) | CCGGGGACTCACAATTCCAAATACTCGAGTATTTGGAATTGTGAGTCCTTTTTG |
|  | Anti-Sense (5’-3’) | AATTCAAAAAGGACTCACAATTCCAAATACTCGAGTATTTGGAATTGTGAGTCC |
| pMIR-LATS1-3’UTR | Forward (5’-3’) | GGACTAGTCCTGTAATGAGGATTTGTAAAAGGGC |
|  | Reverse (5’-3’) | CGACGCGTCGTATTATCCCAGCAAACATTACACTA |
| pMIR-LATS2-3’UTR | Forward (5’-3’) | GGACTAGTCCCACCCCCACCACTCGCTGCCTCCCA |
|  | Reverse (5’-3’) | CGACGCGTCGGTGCCAGTAGAAGCTTTTCAAAGGTAA |
