# Supplementary figures and images for: STARD13-correlated ceRNA network-directed inhibition on YAP/TAZ activity suppresses stemness of breast cancer via co-regulating Hippo and Rho-GTPase/F-actin signaling
Source: J Hematol Oncol. 2018 May 30;11:72. doi: 10.1186/s13045-018-0613-5 (PMC5977742; doi:10.1186/s13045-018-0613-5)

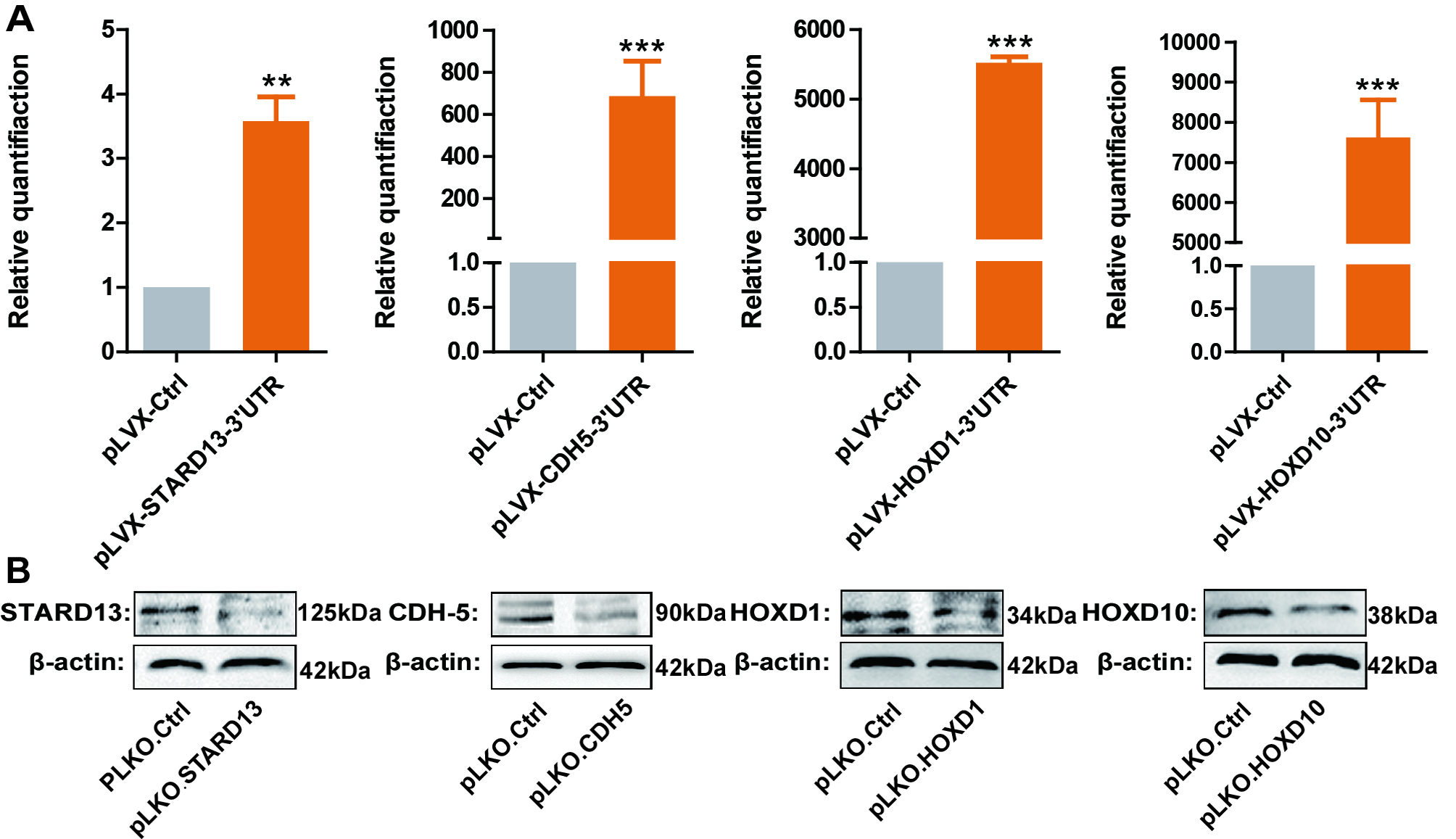

Supplement: Supplementary file 5 — Figure S1. The infection efficiency of lentivirus. (A) Lentiviral infection efficiency of MDA-MB-231 cells stably expressing STARD13-3′UTR, CDH5-3′UTR, HOXD1-3′UTR, and HOXD10-3′UTR was examined by qRT-PCR. (B) Lentiviral infection efficiency of MCF-7 cells stably depleted of STARD13, CDH5, HOXD1, and HOXD10 was verified by Western blot analysis. Data were presented as the mean ± SD, n = 3, ***p < 0.001 vs. Ctrl. (TIF 1373 kb) [file 13045_2018_613_MOESM5_ESM.tif]

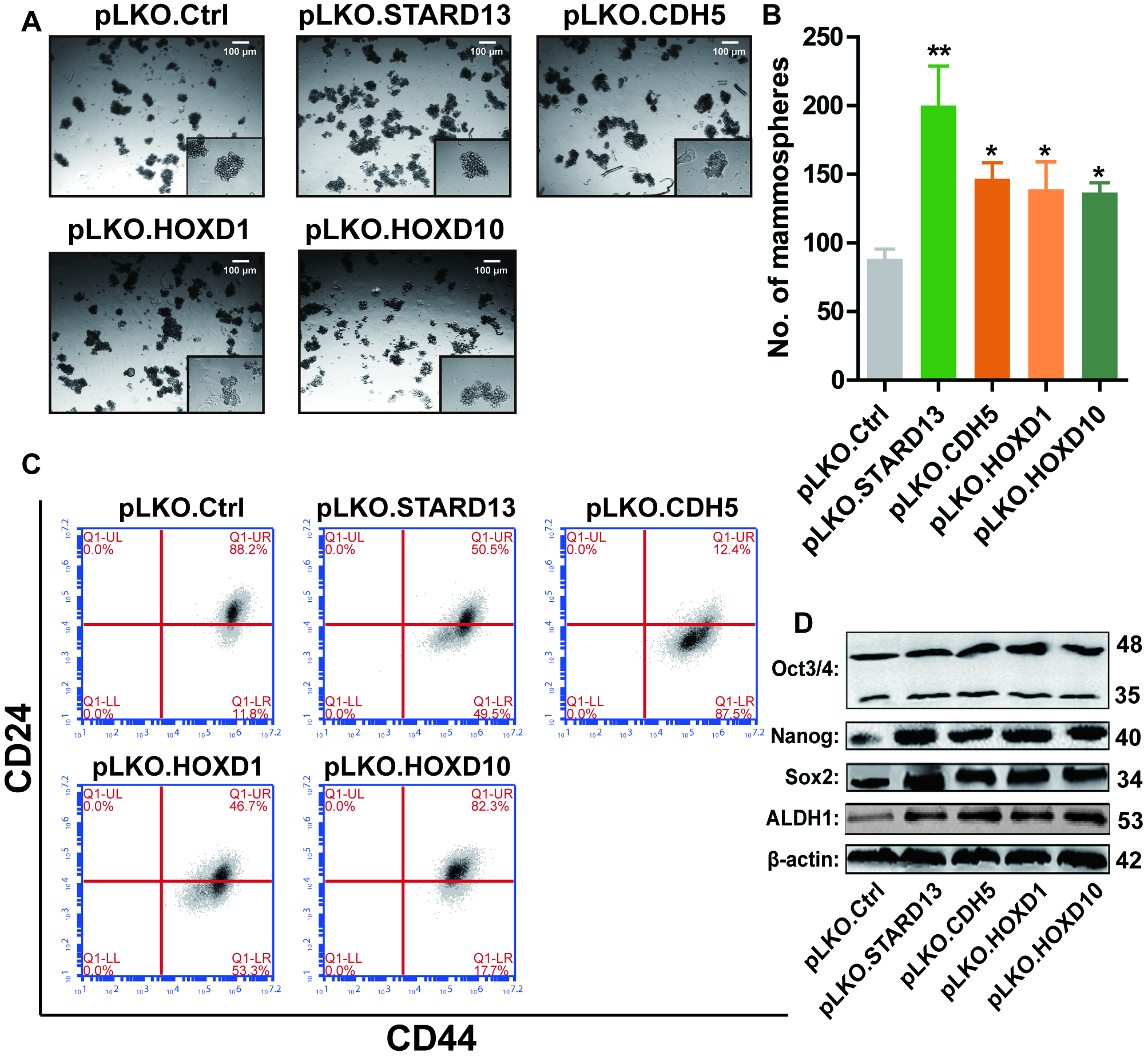

Supplement: Supplementary file 6 — Figure S2. MCF-7 cells depleted of STARD13-correlated ceRNAs gain CSC traits. (A) Phase contract images of mammospheres formed by MCF-7 cells with STARD13-correlated ceRNA knockdown. (B) Quantification of mammospheres formed in (A). (C) Represented FACS profile of MCF-7 cells described in (A). (D) Identification of stemness-related genes expression (ALDH1, OCT4, and Nanog) by Western blot analysis in MCF-7 cells described in (A). Data were presented as the mean ± SD, n = 3, *p < 0.05, **p < 0.01 vs. pLVX-Ctrl. (TIF 3016 kb) [file 13045_2018_613_MOESM6_ESM.tif]

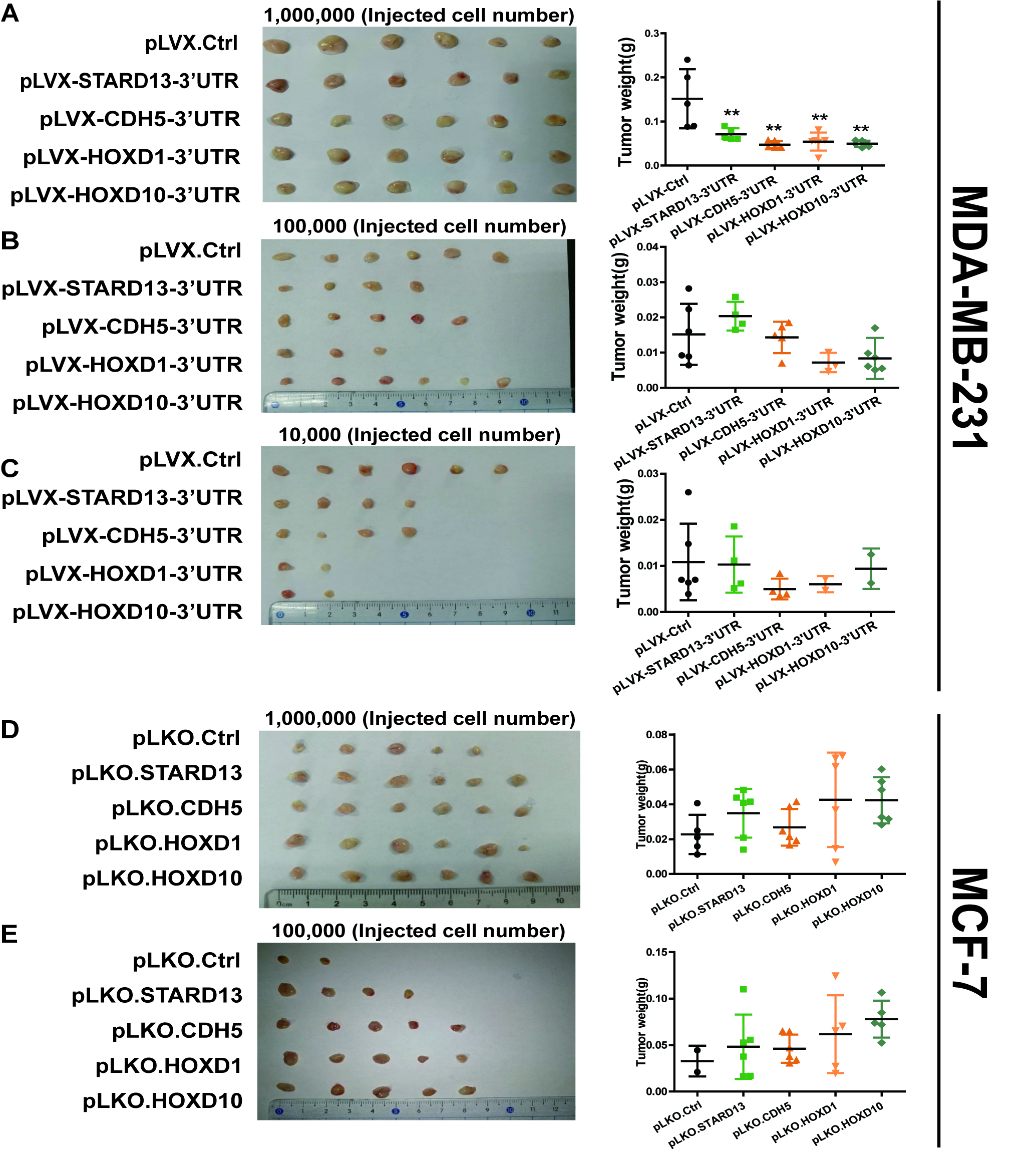

Supplement: Supplementary file 7 — Figure S3. STARD13-correlated ceRNA network inhibits CSC traits of breast cancer cells in vivo. (A, B, and C) Images (left) and weight (right) of tumors harvested when serially diluted MDA-MB-231 cells with STARD13-correlated ceRNAs-3′UTR overexpression were planted. (D and E) Images (left) and weight (right) of tumors harvested when serially diluted MCF-7 cells with STARD13 or its ceRNA knockdown were planted. (TIF 4230 kb) [file 13045_2018_613_MOESM7_ESM.tif]

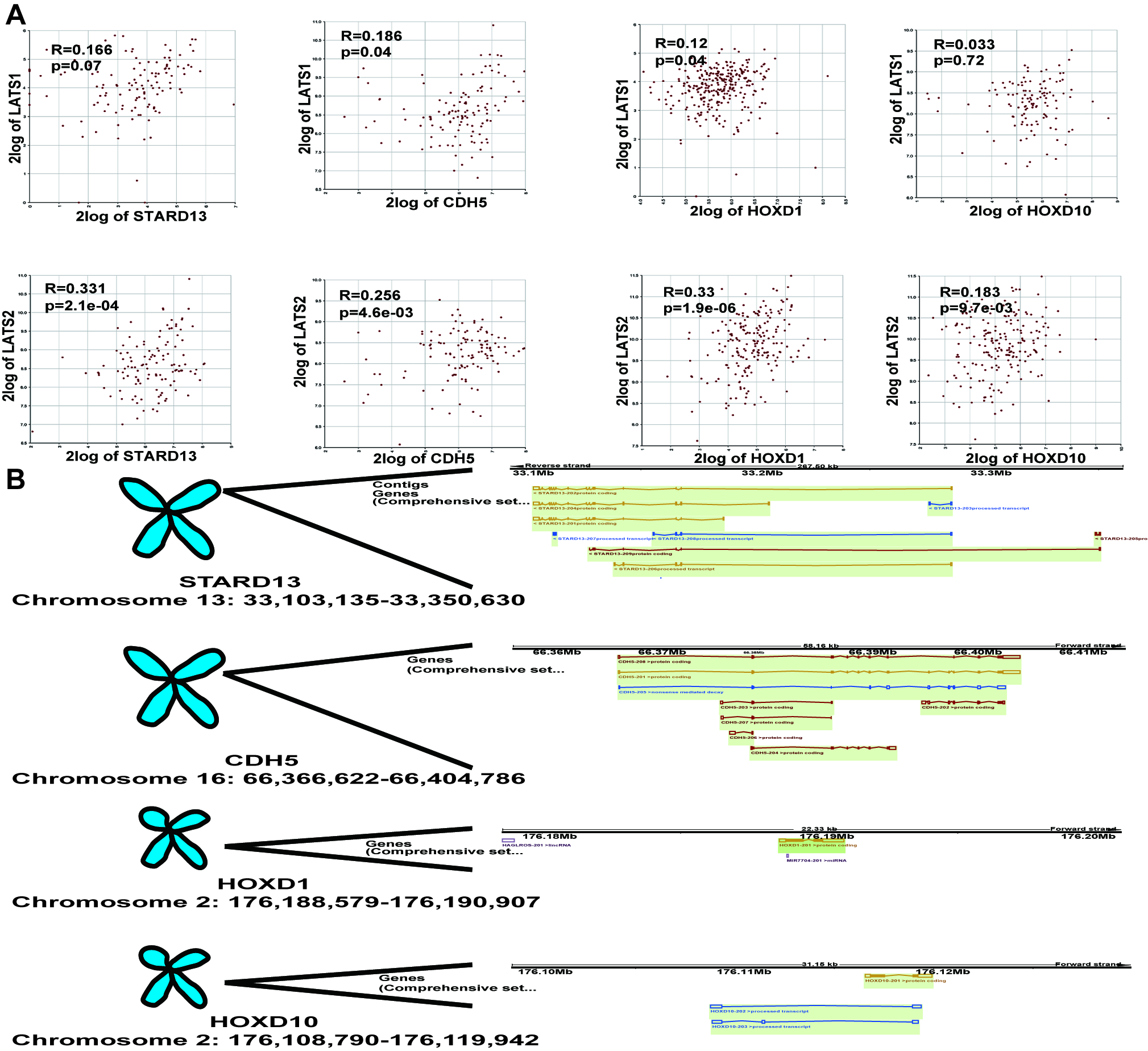

Supplement: Supplementary file 8 — Figure S4. (A) Correlation analysis between LATS1/2 and STARD13, CDH5, HOXD1, and HOXD10, based on the microarray data downloaded from the TCGA data portal. (B) The ceRNA sequence and the genomic locus. (TIF 1525 kb) [file 13045_2018_613_MOESM8_ESM.tif]

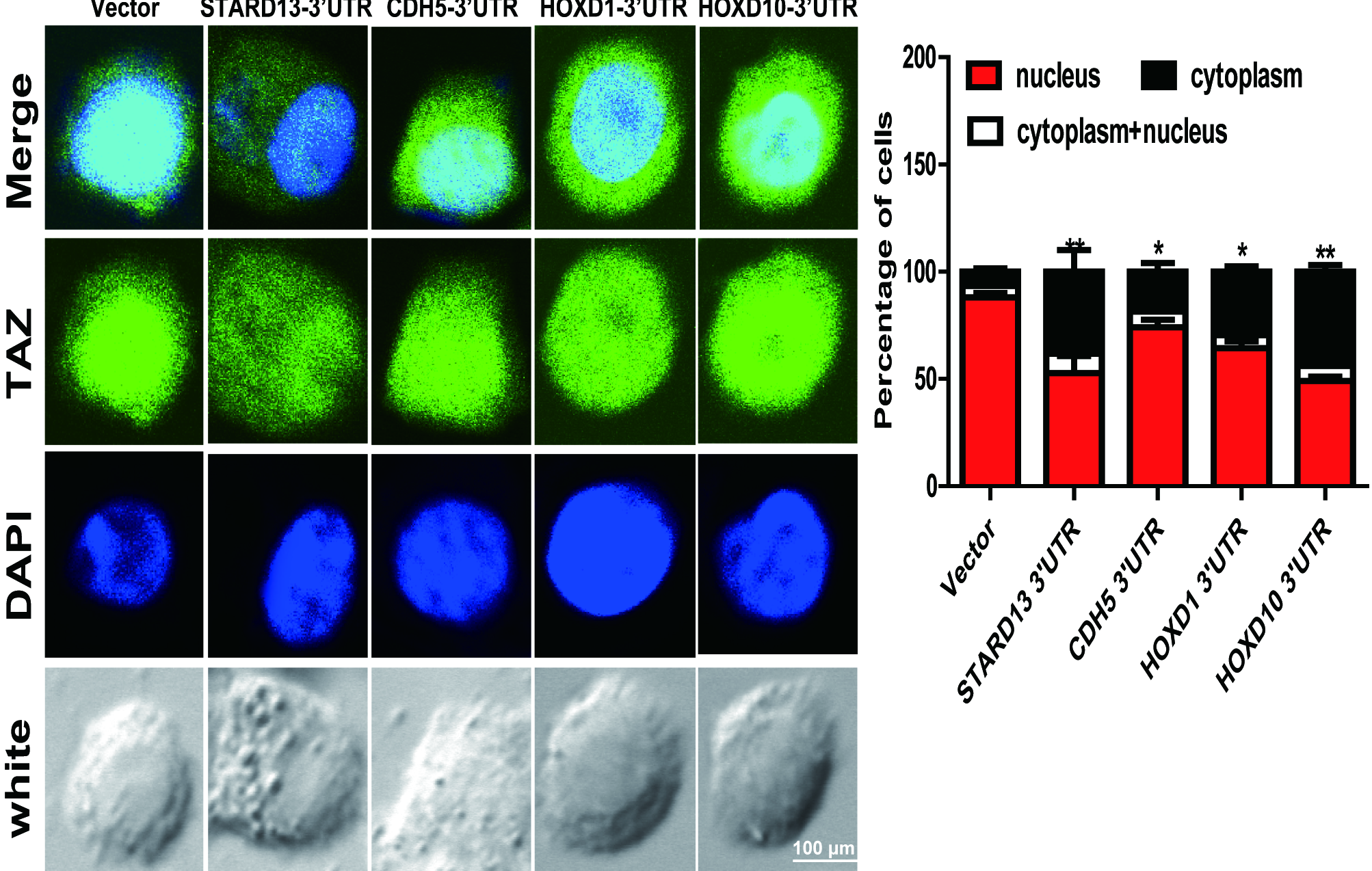

Supplement: Supplementary file 9 — Figure S5. STARD13-correlated ceRNAs-3′UTRs regulate TAZ nuclear abundance. Confocal images of TAZ distribution in MDA-MB-231 cells with STARD13-correlated ceRNAs-3′UTR overexpression or not. Data were presented as the mean ± SD, n = 3, *p < 0.05, **p < 0.01 vs. Vector. (TIF 4364 kb) [file 13045_2018_613_MOESM9_ESM.tif]

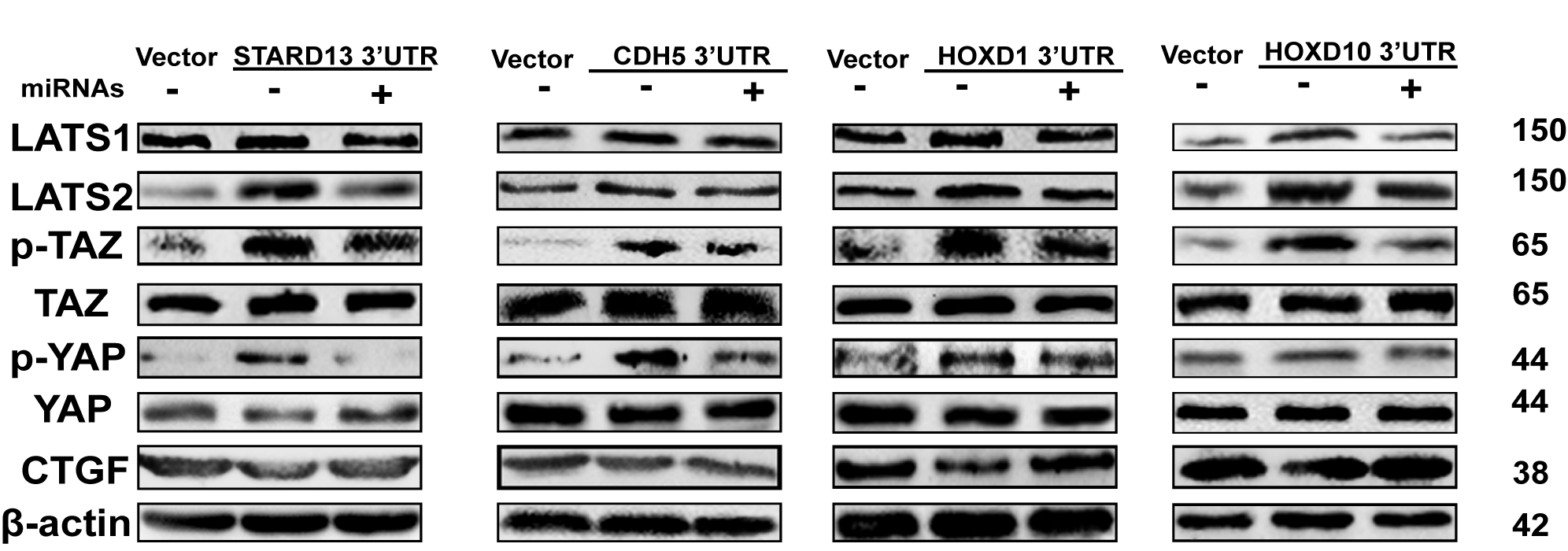

Supplement: Supplementary file 11 — Figure S6. Target miRNAs attenuated the promotive effects of STARD13-correlated ceRNA network on Hippo signaling. Target miRNAs (miR-424, miR-374a, miR-590-3p, miR-448, and miR-15a) mimics mix was co-transfected with STARD13-correlated ceRNAs-3′UTR overexpression constructs or not, the protein level of LATS1/2 and downstream effectors (p-YAP/p-TAZ, YAP/TAZ, and CTGF) was examined. (TIF 1437 kb) [file 13045_2018_613_MOESM11_ESM.tif]

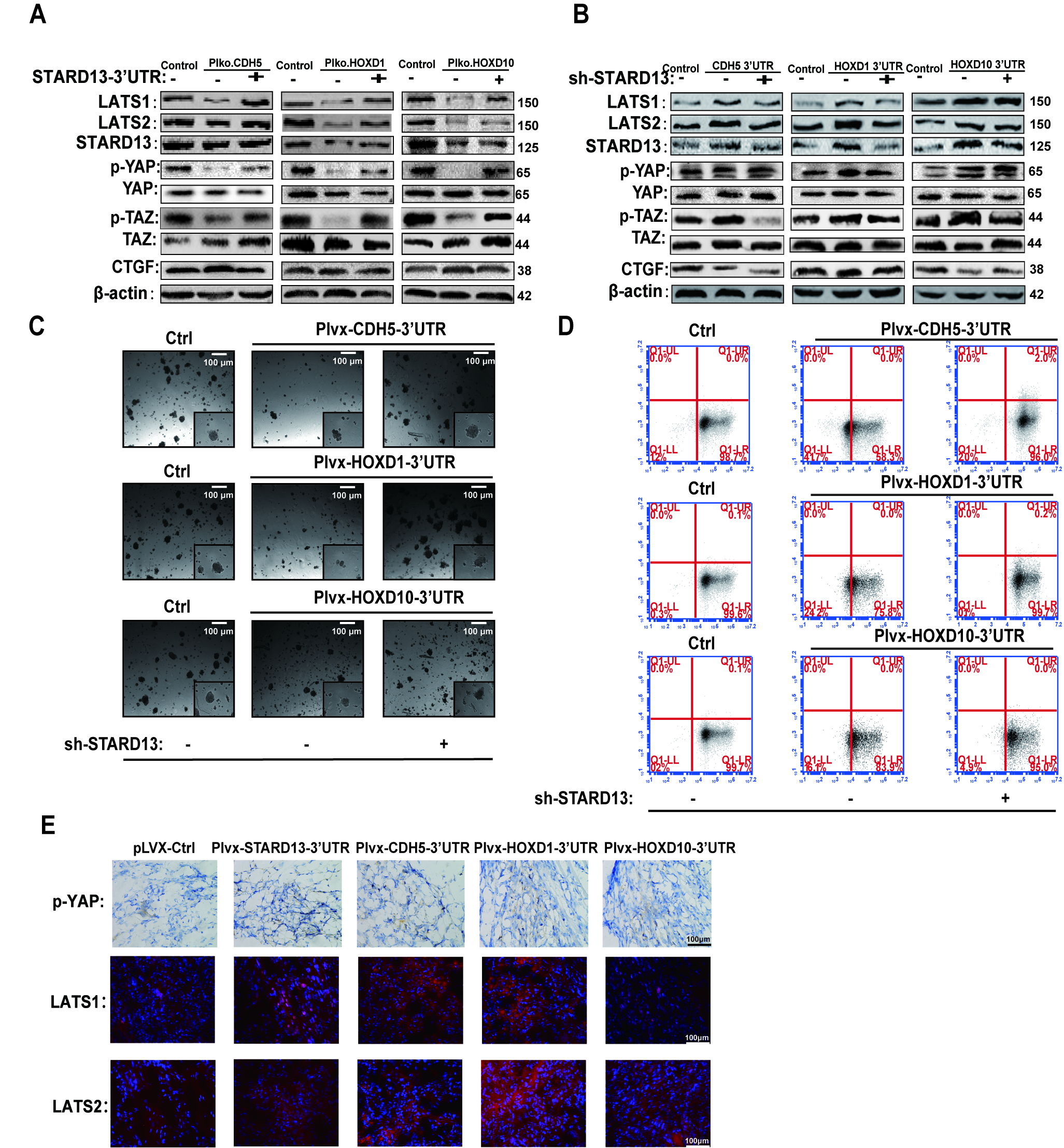

Supplement: Supplementary file 12 — Figure S7. CDH5, HOXD1, HOXD10-3′UTRs regulate Hippo signaling and CSC traits of breast cancer cells through STARD13. (A) Western blot analysis of lysates from MCF-7 cells with its ceRNA knockdown plus STARD13 3′UTR co-transfection or not. (B) Western blot analysis of lysates from MDA-MB-231 cells with STARD13 ceRNAs-3′UTR overexpression plus STARD13 knockdown or not. (C and D) Phase contract images of mammospheres (C) formed by MDA-MB-231 cells described in (A). Representative FACS profile (D) of them with CD24− and CD44+ markers by flow cytometry analysis. (E) Represented images of p-YAP, LATS1, and LATS2 staining of tumors harvested when 1,000,000 cells were injected in Fig. 2a. (TIF 5010 kb) [file 13045_2018_613_MOESM12_ESM.tif]

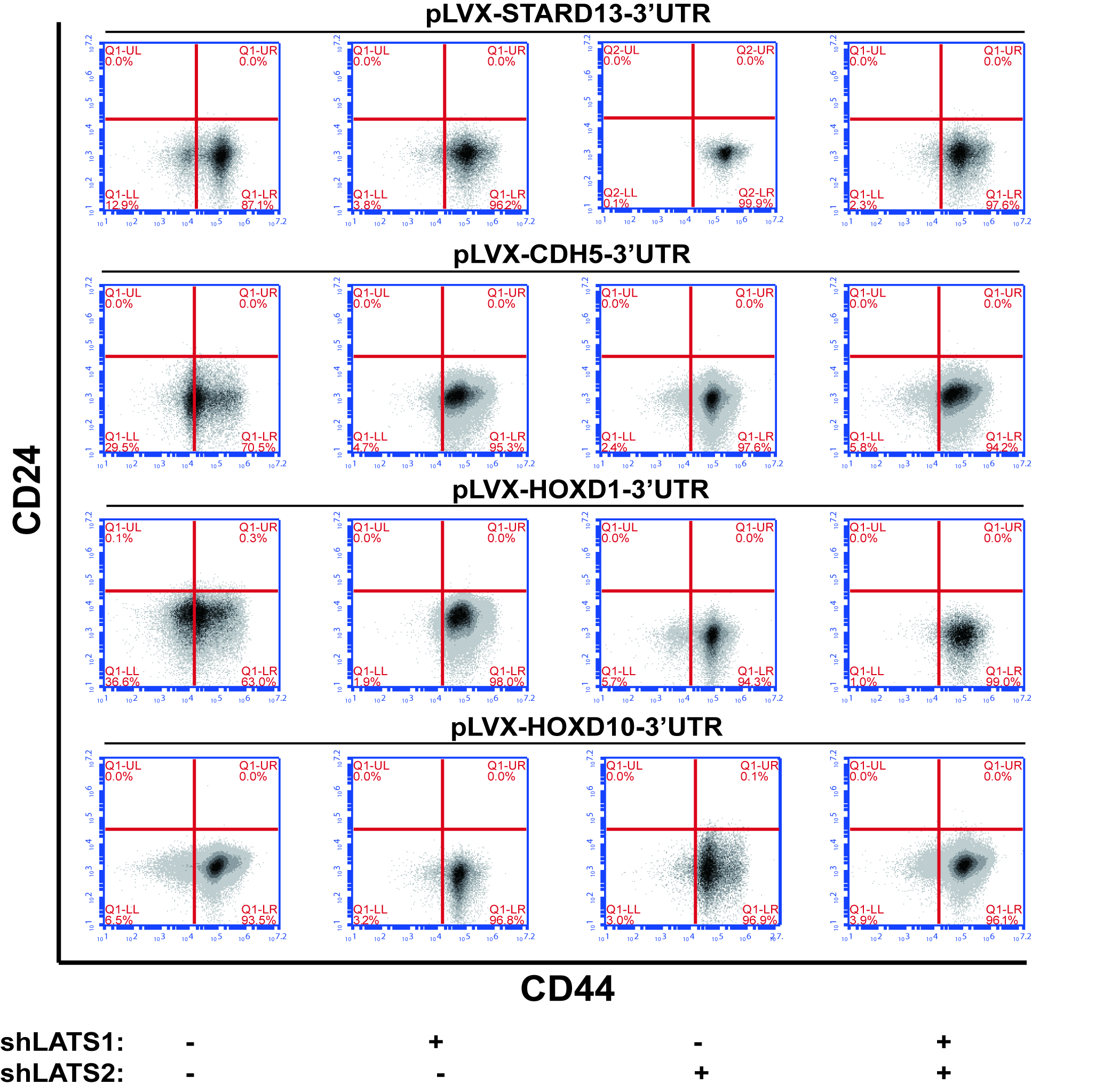

Supplement: Supplementary file 13 — Figure S8. Representative FACS profile of MDA-MB-231 cells with STARD13- or its ceRNAs-3′UTR overexpression plus LATS1 or LATS2 or LATS1/2 knockdown by lentiviral infection. (TIF 1568 kb) [file 13045_2018_613_MOESM13_ESM.tif]

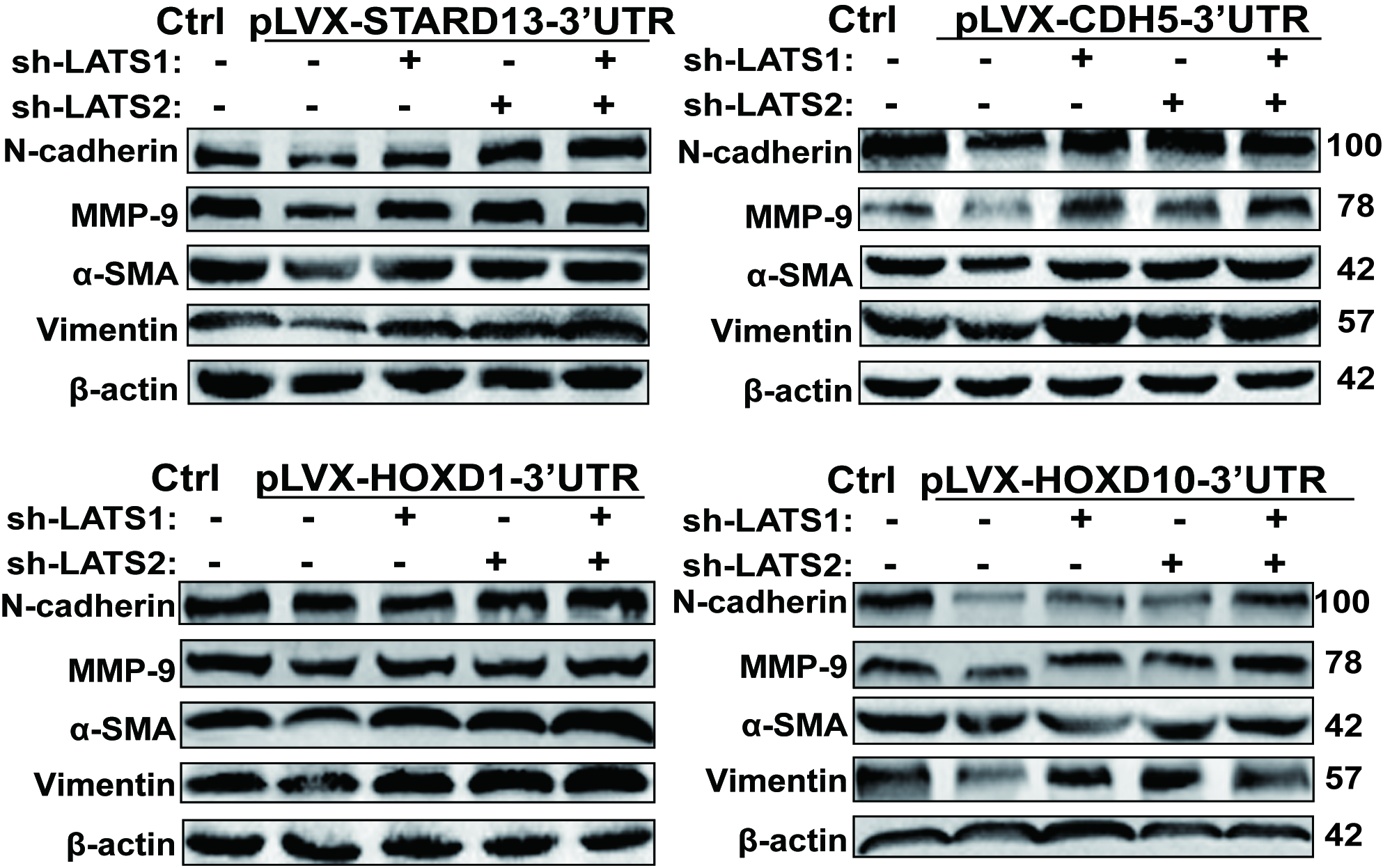

Supplement: Supplementary file 14 — Figure S9. STARD13-correlated ceRNA network regulates breast cancer EMT through LATS1/2. EMT marker (see in main text) expressions were measured in MDA-MB-231 cells with STARD13- or its ceRNAs-3′UTR overexpression plus LATS1 or LATS2 or LATS1/2 knockdown by lentiviral infection. (TIF 2846 kb) [file 13045_2018_613_MOESM14_ESM.tif]

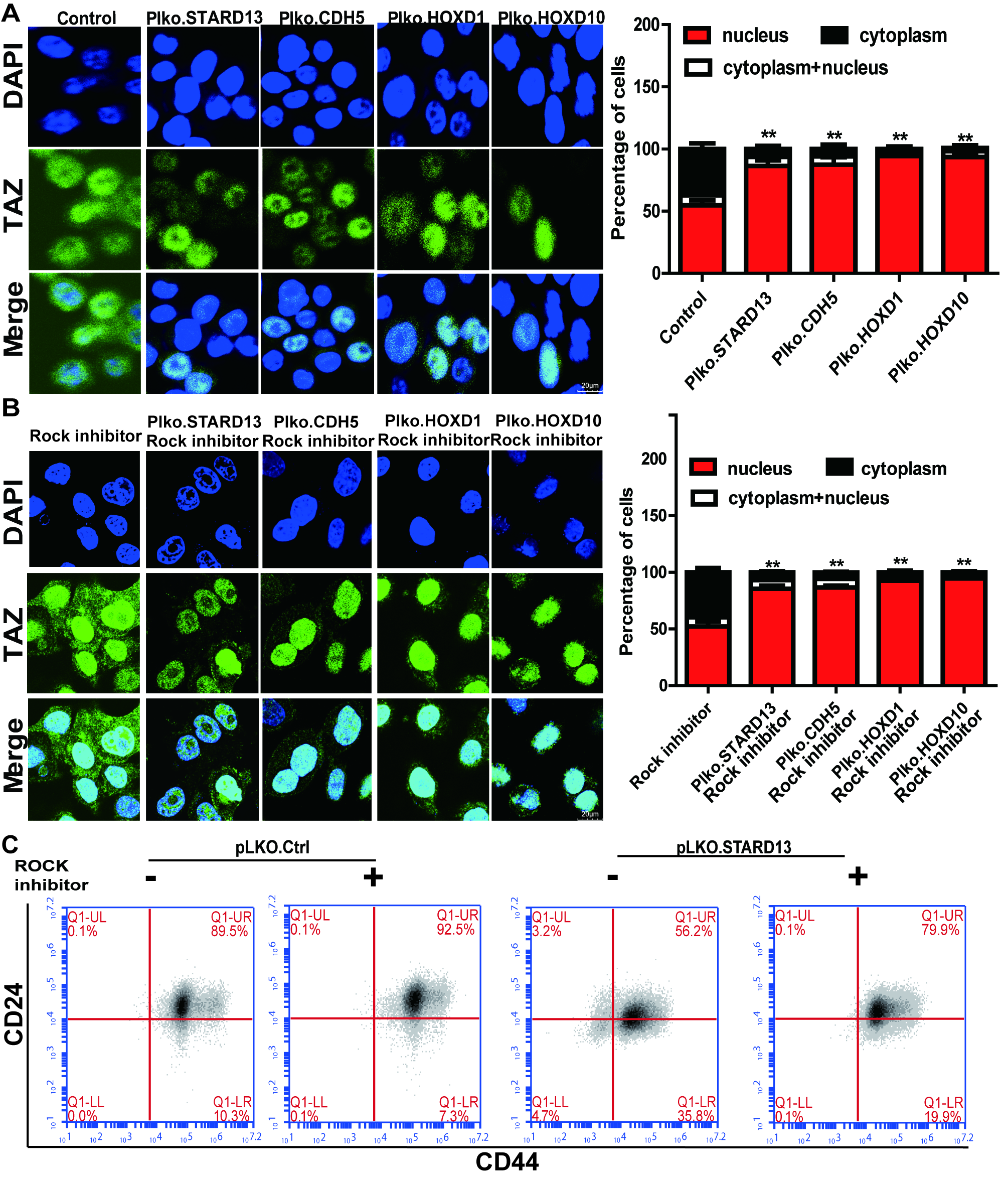

Supplement: Supplementary file 15 — Figure S10. STARD13-correlated ceRNA network regulate TAZ nuclear abundance by inhibiting RhoA-ROCK signaling. (A) Confocal images of TAZ distribution in MCF-7 cells with STARD13-correlated ceRNA knockdown. (B) Confocal images of TAZ distribution in MCF-7 cells with STARD13-correlated ceRNA knockdown plus ROCK inhibitor (Y-27632) treatment or not. (C) Representative FACS profile of MCF-7 cells with STARD13 knockdown plus ROCK inhibitor treatment. Data were presented as the mean ± SD, n = 3, *p < 0.05, **p < 0.01 VS. Control or Rock inhibitor. (TIF 5007 kb) [file 13045_2018_613_MOESM15_ESM.tif]

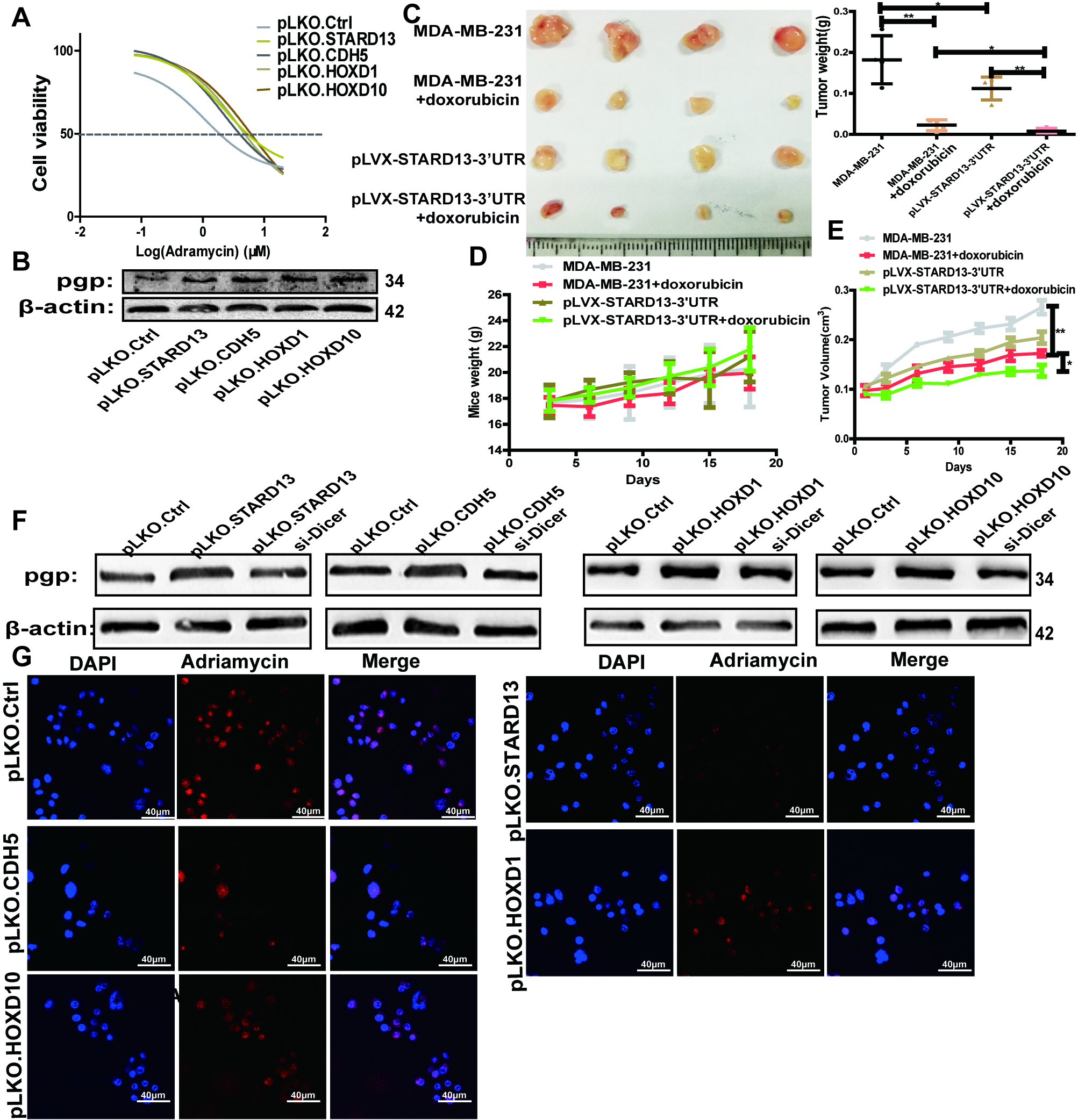

Supplement: Supplementary file 16 — Figure S11. Depletion of STARD13-correlated ceRNAs dampens the response of breast cancer cells to doxorubicin. (A) IC50 curves of MCF-7 cells with STARD13-correlated ceRNA knockdown and were fitted with a nonlinear regression model and were presented as log (Doxorubicin) vs cell viability. (B) Western blot assay of lysates from MCF-7 cells with STARD13-correlated ceRNA knockdown. (C) Images of tumors harvested when STARD13 3′UTR stable overexpression cells were planted and followed by doxorubicin treatment or not. The weight of tumors harvested in (C) was monitored. (D) The weight of mice depicted in (C) was monitored. (E) The volume of tumors harvested in (C) was monitored. (G) Confocal images of MCF-7 cells described in (B) with doxorubicin treatment. Depletion of STARD13-correlated ceRNAs impaired the cellular retention of doxorubicin. (F) Western blot assay of lysates from MCF-7 cells with STARD13-correlated ceRNA knockdown plus si-Dicer or not. (TIF 3999 kb) [file 13045_2018_613_MOESM16_ESM.tif]
