# Supplementary material for: STARD13-correlated ceRNA network-directed inhibition on YAP/TAZ activity suppresses stemness of breast cancer via co-regulating Hippo and Rho-GTPase/F-actin signaling
Source: J Hematol Oncol. 2018 May 30;11:72. doi: 10.1186/s13045-018-0613-5 (PMC5977742; doi:10.1186/s13045-018-0613-5)
Supplement: Supplementary file 10 — Table S5. The number of common miRNA binding sites on STARD13 3′UTR and LATS1/2 3′UTR is predicted using Targetscan 6.2 and microRNA.org. (DOC 33 kb) [file 13045_2018_613_MOESM10_ESM.doc]

**Additional file 10: Table S5. The number of common miRNA binding sites on STARD13 3'UTR and LATS1/2 3'UTR are predicted using Targetscan 6.2 and microRNA.org.**

|  | STARD13 | LATS1 | LATS2 |
| --- | --- | --- | --- |
| miR-590-3p | 4 | 2 | 4 |
| miR-200a/b/c | 1 | 1 | 1 |
| miR-429 | 1 | 1 | 1 |
| miR-211 | 3 | 1 | 1 |
| miR-448 | 3 | 1 | 3 |
| miR-10b | 1 | 1 | 1 |
| miR-15a/b | 2 | 1 | 2 |
| miR-16 | 2 | 1 | 2 |
| miR-195 | 2 | 1 | 2 |
| miR-424 | 2 | 1 | 2 |
| miR-374a/b | 2 | 1 | 4 |
| miR-153 | 3 | 1 | 0 |
